# Antimicrobial Activity of a Library of Thioxanthones and Their Potential as Efflux Pump Inhibitors

**DOI:** 10.3390/ph14060572

**Published:** 2021-06-15

**Authors:** Fernando Durães, Andreia Palmeira, Bárbara Cruz, Joana Freitas-Silva, Nikoletta Szemerédi, Luís Gales, Paulo Martins da Costa, Fernando Remião, Renata Silva, Madalena Pinto, Gabriella Spengler, Emília Sousa

**Affiliations:** 1 Laboratory of Organic and Pharmaceutical Chemistry, Department of Chemical Sciences, Faculty of Pharmacy, University of Porto, Rua de Jorge Viterbo Ferreira, 228, 4050-313 Porto, Portugal; fduraes5@gmail.com (F.D.); apalmeira@ff.up.pt (A.P.); madalena@ff.up.pt (M.P.); 2 CIIMAR-Interdisciplinary Centre of Marine and Environmental Research, University of Porto, Novo Edifício do Terminal de Cruzeiros do Porto de Leixões, Avenida General Norton de Matos, S/N, 4450-208 Matosinhos, Portugal; joanafreitasdasilva@gmail.com (J.F.-S.); pmcosta@icbas.up.pt (P.M.d.C.); 3 UCIBIO-REQUIMTE, Laboratory of Toxicology, Faculty of Pharmacy, University of Porto, Rua de Jorge Viterbo Ferreira 228, 4050-313 Porto, Portugal; barbara_cruz96@hotmail.com (B.C.); remiao@ff.up.pt (F.R.); rsilva@ff.up.pt (R.S.); 4 ICBAS–Institute of Biomedical Sciences Abel Salazar, Universidade do Porto, Rua de Jorge Viterbo Ferreira 228, 4050-313 Porto, Portugal; 5 Department of Medical Microbiology, Albert Szent-Györgyi Health Center and Faculty of Medicine, University of Szeged, Semmelweis utca 6, 6725 Szeged, Hungary; szemeredi.nikoletta@med.u-szeged.hu; 6 Department of Molecular Biology, ICBAS–Instituto de Ciências Biomédicas Abel Salazar, University of Porto, Rua de Jorge Viterbo Ferreira 228, 4050-313 Porto, Portugal; lgales@ibmc.up.pt; 7 Bioengineering & Synthetic Microbiology, I3S–Instituto de Investigação e Inovação em Saúde, University of Porto, Rua Alfredo Allen 208, 4200-135 Porto, Portugal

**Keywords:** thioxanthones, antimicrobial resistance, efflux pumps, biofilm, quorum-sensing

## Abstract

The overexpression of efflux pumps is one of the causes of multidrug resistance, which leads to the inefficacy of drugs. This plays a pivotal role in antimicrobial resistance, and the most notable pumps are the AcrAB-TolC system (AcrB belongs to the resistance-nodulation-division family) and the NorA, from the major facilitator superfamily. In bacteria, these structures can also favor virulence and adaptation mechanisms, such as quorum-sensing and the formation of biofilm. In this study, the design and synthesis of a library of thioxanthones as potential efflux pump inhibitors are described. The thioxanthone derivatives were investigated for their antibacterial activity and inhibition of efflux pumps, biofilm formation, and quorum-sensing. The compounds were also studied for their potential to interact with P-glycoprotein (P-gp, ABCB1), an efflux pump present in mammalian cells, and for their cytotoxicity in both mouse fibroblasts and human Caco-2 cells. The results concerning the real-time ethidium bromide accumulation may suggest a potential bacterial efflux pump inhibition, which has not yet been reported for thioxanthones. Moreover, in vitro studies in human cells demonstrated a lack of cytotoxicity for concentrations up to 20 µM in Caco-2 cells, with some derivatives also showing potential for P-gp modulation.

## 1. Introduction

Multidrug resistance (MDR), the ability to evade the activity of a wide range of structurally diverse compounds, is a rising problem in treating a multiplicity of pathologies, such as cancer and infectious diseases [1]. One of the reasons for this phenomenon is the overexpression of efflux pumps, transmembrane structures present in every cell from human to bacteria, which usually play an important part in detoxification pathways, but are also responsible for the appearance of MDR [1,2]. In many cancers, overexpression of P-glycoprotein (P-gp, ABCB1) contributes to the found multidrug resistance (MDR) phenotype [1,2]. P-gp has been an interesting target to reverse MDR in cancer, although attempts to find successful therapies have been challenging [3]. Bacteria present a similar resistance mechanism, resorting to the overexpression of efflux pumps, which leads to an increased outward transport of drugs, rendering them ineffective [4]. These are divided into six families, and of these, the resistance-nodulation-division (RND) [5] and the major facilitator superfamily (MFS) [6] are the most relevant. One of the most notable pumps in the RND family, well-distributed in Gram-negative bacteria, is the AcrAB-TolC efflux system, comprised of three distinct parts: AcrA, the membrane fusion protein; AcrB, a resistance-nodulation-division transporter; and TolC, a multifunctional outer membrane channel [4]. The MFS efflux pumps are more associated with Gram-positive bacteria, and the NorA pump acquires the most relevance in this family [4]. In bacteria, efflux pumps are associated with many other mechanisms, such as resistance, adaptation, and virulence mechanisms. When it comes to biofilm, the capacity of certain microorganisms to form a complex association that latches onto surfaces, efflux pumps have been proven to play a pivotal role [7]. They can be involved in the efflux of quorum-sensing molecules and/or extracellular polymeric substances, which regulate bacteria communication and the development of the biofilm matrix, respectively. These pumps are also responsible for the efflux of harmful molecules and can influence the aggregation [8,9].

Thioxanthones are compounds that possess a dibenzo-γ-pyrone, tricyclic scaffold, they are privileged structures [10] and they have previously been described as capable of modulating the human efflux pump P-gp [11]. Thioxanthones have also demonstrated their potential as antimicrobials and have proved to be effective in enhancing the activity of antibiotics in resistant bacterial strains [12]. They are isosteres of xanthones, which have recently been reported as bacterial efflux pump inhibitors [13]. Herein, a library of thioxanthones as potential efflux pump inhibitors was designed and synthesized, and characterized according to their antimicrobial activity, synergy with antibiotics, bacterial efflux pump inhibition and related resistance mechanisms, such as inhibition of biofilm formation and quorum-sensing. Additionally, studies on the ability of new thioxanthones to modulate mammalian P-gp were also carried out. Docking studies were performed to understand the interactions between the hit compounds with the best overall results and putative efflux pump targets. Results obtained suggest the potential of these compounds to tackle multiple bacterial resistance mechanisms.

## 2. Results and Discussion

### 2.1. Chemistry

The aim of this work was to build a library of thioxanthone derivatives with potential for bacterial efflux pump inhibition, based on previously found P-gp inhibitors [11]. Moieties involved in bacterial efflux pump inhibitors and antimicrobials were associated to a thioxanthone scaffold, previously explored by some of us to discover P-gp modulators [11]. Firstly, with a preliminary virtual screening, docking studies were performed to select the thioxanthones that preferably interacted with bacterial efflux pumps (Section 2.3). Thirteen thioxanthones and four tetracyclic thioxanthenes were selected and synthesized from commercially available 1-chloro-4-propoxy-9*H*-thioxanthen-9-one (**1**, Table 1). The synthesis of compounds **2**–**7** (Table 1) was performed as previously described [11]. New 1-nitrogen substituted thioxanthones **8**–**13** (Table 1) were synthesized in a similar way, via a copper-catalyzed Ullmann-type C–N coupling (procedure **a**). The selection of chemical ligands to couple in order to obtain the proposed derivatives included primary or secondary aliphatic amines and sulfamides. Compound **4**, a 1-methoxyl thioxanthone derivative, was a secondary product of the reaction in procedures **a** and **b**, further included in the screening. A 1-bromine derivative (**14**, Table 1) was also synthesized, based on the reaction described by Grossman et al. (2006), starting from **1** (procedure **c**) [14], and was also used as a starting material for the coupling with sulfamides, which were less reactive (procedure **b**). Four tetracyclic thioxanthenes (**15**–**18**, Table 1) were also synthesized under the same conditions as compounds **8**–**13**. These were obtained from guanidine or urea derivatives leading to a quinazoline-thiochromene scaffold, as previously described [15].

### 2.2. Structure Elucidation

The structures of the compounds were elucidated by nuclear magnetic resonance (NMR), Fourier-transform infrared spectroscopy (FT-IR), high resolution mass spectrometry (HRMS) and, in the case of compounds **13**–**16,** X-ray crystallography. Figure S1 (Supplementary Data) presents the main ^1^H and ^13^C NMR chemical shifts of the signals for the newly synthesized thioxanthones **8**–**14**.

The newly synthesized thioxanthones **8**–**14** displayed characteristic signals of the thioxanthone skeleton in the NMR spectra, previously described for compounds **2**–**7** [11]. The heteronuclear single quantum correlation (HSQC) and heteronuclear multiple bond correlation (HMBC) spectra were also obtained. HMBC was particularly useful in the quaternary carbons assignment, as illustrated in Figure S2 (Supplementary Data).

**Figure 1:**
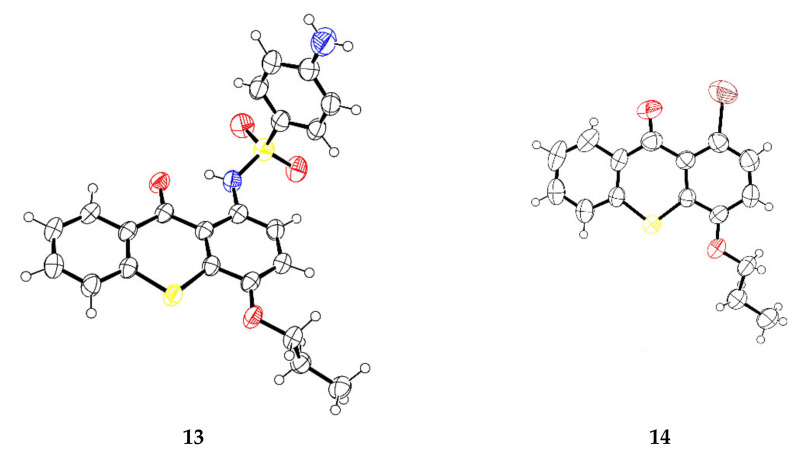
ORTEP view of compounds **13** and **14**.

The structures of compounds **13** and **14** were determined by single crystal X-ray diffraction (Figure 1). A general feature of xanthones [16], which is shared by **13** and **14**, is the planarity of the three ring skeleton. The central pyranoid rings have partial aromatic character, with the shortening of the C—S bonds showing the partial double-bond character (ranging from 1.741(4) to 1.752(3) Å in **13** and **14**, while C—S single bond lengths in tetrahydrothio-γ-pyrone are 1.806(4) and 1.814(4)Å [17]).

### 2.3. Docking Studies

The library of 13 thioxanthone derivatives and four tetracyclic thioxanthenes was investigated, to find their predicted binding affinities against relevant bacterial efflux pumps. Due to the effect of xanthones on strains with these transporters [13], and due to structurally related thioxanthenes reported as NorA inhibitors [18], we hypothesized that the series of in-house thioxanthones **2**–**7**, with P-glycoprotein inhibitory activity [11], as well as the newly synthesized thioxanthones **8**–**18** could be effective efflux pump inhibitors. These derivatives were docked against the most relevant targets, the AcrAB-TolC efflux system from the RND family [19], and the MFS pump NorA [20], and their predicted affinity against these targets was ranked. To predict their selectivity for bacterial efflux pumps, docking studies against mammalian P-glycoprotein were also performed. Both NorA and human P-gp do not have a crystal structure deposited in the Protein Data Bank, and homology models needed to be built. Reference compounds described as efflux pump inhibitors were used as positive controls.

Docking studies were performed based on the crystal structures of the AcrB (4DX5), AcrA (2F1M), and TolC (1EK9) portions of the AcrAB-TolC efflux system. For AcrB and AcrA, these studies were performed in two different sites: the substrate-binding site (SBS) and the hydrophobic trap (HT) for AcrB [21], and the helical hairpin (HH) and the lipoyl domain (LD) for AcrA [19]. For TolC, only the lysine residues that interact with the 3,3′-dithiobis(sulfosuccinimidyl proprionate) (DTSSP) bifunctional crosslinker [19] were considered. For the NorA homology model, the sites used for docking of the compounds were the binding core region (BCR) and the cytoplasmic side (CS), as described in [22]. Additionally, compounds **2**–**18** were docked into a homology model of the mammalian efflux pump P-gp, in the transmembrane domain (TMD) and in the nucleotide binding domain (NBD) [11]. The results are presented in Table 2.

The results predicted thioxanthones **2**–**18** to bind with an affinity similar to the reported inhibitors, considering that they present comparable docking scores, and globally they present better scores than the starting materials **1** and **14**. Among the portions of the AcrAB-TolC efflux system, compounds generally present the highest affinities towards the SBS of AcrB portion, and AcrA would be the portion towards which the compounds are predicted to present the least affinity, only with better docking scores than for the hydrophobic trap of AcrB. In terms of docking into the NorA homology model, there is an even distribution between both sites targeted. It is noteworthy that most of the compounds presented the lowest docking scores for the P-gp model, compared to all the bacterial pumps tested, suggesting that these compounds also act as mammalian efflux substrates (with the exception of compounds **8** and **10**). Considering that the compounds presented docking scores similar to those previously reported as bacterial efflux pump inhibitors, this library was selected to be investigated *in vitro* in resistant bacterial models. 

### 2.4. Antibacterial Activity and Synergy with Antimicrobials

The referred library of thioxanthones was initially tested for their antimicrobial activity against six bacterial strains: the Gram-negative *Escherichia coli* ATCC 25922, *Pseudomonas aeruginosa* ATCC 27853 and *Salmonella enterica* serovar Typhimurium SL1344 (SE03), with the *acrA* gene deleted, and the Gram-positive *Staphylococcus aureus* ATCC 29213, *Enterococcus faecalis* ATCC 29212 and the methicillin and ofloxacin-resistant clinical isolate *S. aureus* 272123. For the synergy assay, two clinically relevant isolates were used: the extended-spectrum β-lactamase (ESBL)-producing *E. coli* SA/2 [23] and the vancomycin-resistant *E. faecalis* (VRE) B3/101 [23]. The antibiotics used were cefotaxime (CTX) and vancomycin (VAN), respectively. 

The results are depicted in Table 3 and show that, out of all the compounds tested, only the aminated derivative **2** and the sulfamide derivative **12** presented activity against the used strains, with a minimum inhibitory concentration (MIC) of 83 µM and 34 µM for *S. aureus* ATCC 23213 and *E. faecalis* ATCC 23121, respectively, with **12** also active against SE03 (MIC = 50 µM). Curiously, the sulfamide precursor of **12**, 4-sulfamoylbenzoic acid, did not display an observable MIC for the concentrations tested (results not shown). Moreover, sulfamide **12** does not contain the para-amino group essential for the antibacterial activity of these *p*-aminobenzoic acid (PABA) antimetabolites [24]. Therefore, we can hypothesize a distinct mechanism of action for this new antibacterial agent. Moreover, compound **12** was found to be more active than the previously described antibacterial thioxanthones [12], and deserves to be further explored.

Concerning the synergy with antimicrobials, four new thioxanthones (**8**, **10**, **12**, and **18**) were able to decrease the MIC of CTX in *E. coli* SA/2 and one reversed the resistance to vancomycin in *E. faecalis* B3/101. In fact, compounds **8**, **10** and **18** caused a 4-fold decrease in the MIC of CTX, while compound **12** caused a 16-fold decrease. Regarding *E. faecalis* B3/101, compound **9** can cause a 16-fold decrease of the MIC of VAN. As *E. coli* SA/2 is an ESBL producer and *E. faecalis* B3/101 had the capacity to modify the peptidoglycan synthesis pathway, it can be hypothesized that the compounds could interact with these targets, but further studies are needed to elucidate the precise mechanisms.

### 2.5. Efflux Pump Inhibition Assay

To investigate the potential of these compounds as efflux pump inhibitors, a preliminary screening of compounds **2**–**18** on two resistant Gram-positive and Gram-negative bacterial strains was performed to investigate the compounds’ capability of modulating the accumulation of the efflux pump substrate ethidium bromide (EB). *S. aureus* 272123 is a clinical strain and was used to compare the activity of thioxanthones with xanthones which were previously tested in the same model [13]. In this strain, the expressions of the *mepA* and *norA* genes were studied in the presence of antimicrobials of natural origin, and the *norA* expression levels did not change [25]. As AcrA was the target with the least binding affinity (Section 2.3), *Salmonella enterica* serovar Typhimurium SE03, with a deletion of the *acrA* gene, was selected as a Gram-negative strain. 

All the compounds, except for compound **12**, were tested at the concentration of 50 µM, since none of them showed antibacterial activity at this concentration. Compound **12** was tested at one-third of its MIC, 17 µM, for SE03, and at 50 µM for *S. aureus* 272123. The relative fluorescence index (RFI) was calculated based on the means of relative fluorescence units, and theses RFIs are depicted in Table 4. Reserpine and carbonyl cyanide 3-chlorophenylhydrazone (CCCP) were used as positive controls for *S. aureus* 272123 and SE03, respectively, at the sub-MIC concentration of 25 µM.

From the analysis of Table 4, it can be noted that **2**, **3**, **7**, **8**, **9**, **10**, **11**, **13**, **14**, **15**, **16**, and **17** can increase the fluorescence in comparison to the positive control. This can be attributed to the inhibition of the efflux of EB, but the fluorescence emitted by the compound itself can also interfere. Therefore, an analysis of the curves of the variation of fluorescence over the course of the assay was performed (data not shown). Additionally, for compounds **2**, **3**, **9**, **10**, **11**, **14**, **15**, **16**, and **17**, which displayed a high/erratic fluorescence pattern over the duration of the assay, an additional fluorescence investigation was performed (Figures S3–S11, Supplementary Data). Out of these compounds, fluorescence was not a determinant factor for **2** and **3**, along with compounds **7**, **8** and **13,** whose behavior during the real-time EB accumulation assay did not suggest the compounds’ fluorescent interference. It can be noticed that some compounds present negative RFI values. This is attributed to the compound displaying a lower extent of EB efflux inhibition than the control, and these compounds were considered ineffective for this purpose.

**Figure 2:**
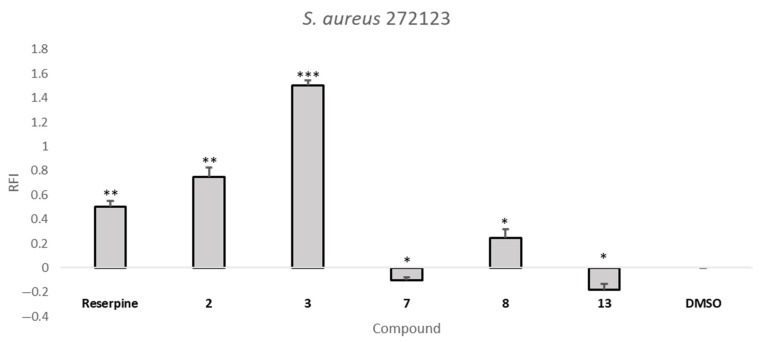

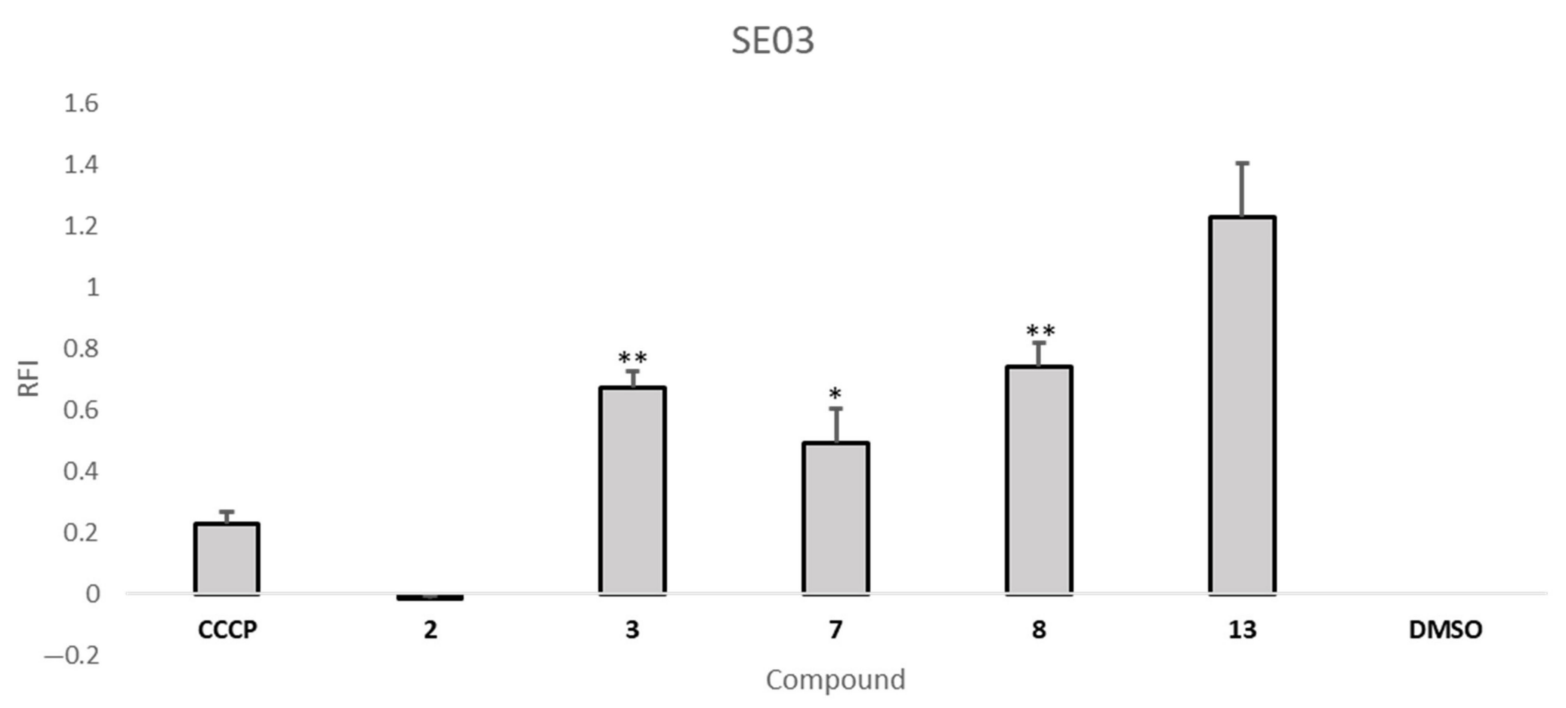
Relative fluorescence index (RFI) of the thioxanthone derivatives **2**, **3**, **7**, **8**, and **13** in *S. aureus* 272123 (**Top**) and *Salmonella enterica* serovar Typhimurium SL1344 (SE03, **Bottom**). Results are presented as mean ± SD. Statistical comparisons were performed using the *t*-test [* *p* < 0.05; ** *p* < 0.01; *** *p* < 0.001 vs. control (DMSO 1% *v*/*v*)].

From the analysis of Figure 2, it can be highlighted that compound **3** is more effective than the positive controls in the inhibition of EB accumulation in both models tested. Compounds **7**, **8**, and **13** were shown to be more effective than CCCP against the efflux systems of SE03, while **2** was found to be more effective than the reference compound reserpine against the efflux systems of the *S. aureus* 272123. None of these compounds present MIC for the respective strain whose efflux they inhibit. This is considered an advantage, as it has been described that an adequate efflux pump should preferably not display antibacterial activity, as this may lead to the appearance of resistance [26].

**Figure 3:**
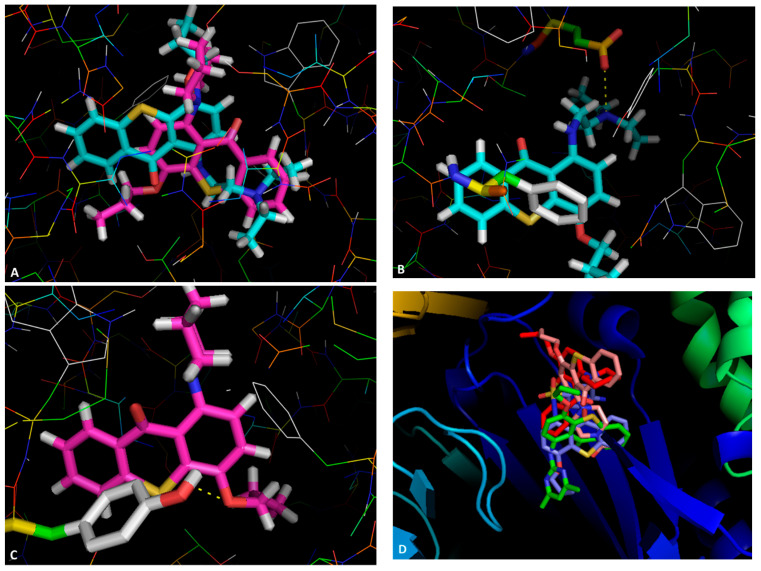
Molecular visualization of thioxanthones on targets in the BCR of the NorA homology model. (**A**) General view of compounds **2** (blue) and **3** (pink); (**B**) Interaction between compound **2** and the BCR; (**C**) Interaction between compound **3** and the BCR; (**D**) in the SBS of AcrB, general view of compounds **3** (red), **7** (pink), **8** (green) and **13** (blue).

The structural similarity of thioxanthones and phenothiazines, previously reported as NorA inhibitors [18], reinforce the idea that thioxanthones may be potential inhibitors towards this target. However, bacteria present multiple efflux pumps of varied families in their membranes, and additional studies would clear the question of which specific pump in being inhibited. To predict how these compounds would interact with their putative targets, a visual inspection of the interactions that could be established was performed using PyMOL. Firstly, compounds **2** and **3** were visualized in a homology model of the NorA pump, particularly in the BCR binding site, as this was the site that was predicted to be where they would bind with the highest affinity (Section 2.3). A general view (Figure 3A) shows these to bind differently, in opposite positions relatively to one another. For compound **2** (Figure 3B), the amine directly bonded to C-1 forms a hydrogen bond with Glu-222 and a π-π stacking interaction between one of the aromatic rings and Phe-47 of the homology model. Compound **3** (Figure 3C) is predicted to interact through the oxygen in the propoxy chain with Tyr-292, establishing a hydrogen bond. The thioxanthene *trans*(*E*)-flupentixol, already described as a NorA inhibitor [18], was also predicted to interact with Tyr-292, but in a different manner, establishing a π-π stacking, and with Trp-293, through hydrogen bonding.

Compounds **3**, **7**, **8** and **13** were visualized in the SBS of AcrB. A general view (Figure 3D) shows that the compounds are predicted to bind in the approximate site, but suggests they interact with different residues. Details of these interactions (Figure S12, Supplementary Data), which could guide the future design of thioxanthones as efflux pump inhibitors, can be found in Supplementary Data.

### 2.6. Biofilm Inhibition and Quorum Sensing Inhibition Assays

Efflux mechanisms are one of the factors that can lead to biofilm-mediated resistance, along with slow growth and reduced penetration due to production of extracellular polysaccharides [27]. It has been demonstrated that efflux pumps can influence the transport of extracellular polymeric substances in biofilms, and regulate the expression of genes concerning biofilm formation, as they have an impact in the way bacterial cells adhere and aggregate to solid surfaces. Additionally, they can also modulate QS, through an influence in the transport of QS signal molecules [28]. Thioxanthones **2–18** were tested for their potential for inhibiting biofilm formation by *S. aureus* ATCC 29213 and the multi-resistant *S. aureus* 272123. The biofilm inhibition, presented in percentage (%), was calculated based on the mean of absorbance units. Reserpine, which has previously been described as both an efflux pump inhibitor and an inhibitor of biofilm formation in Gram-positive bacteria [27,28] was used as control in both strains. The results obtained are presented in Table 5.

From the analysis of the results, it can be seen that compounds **2**, **3** and **13** were active as inhibitors of biofilm formation along with **15** and **16**. Most are preferably inhibitors on *S. aureus* 272123, with better results than reserpine and with compound **3** being the most active derivative. Compound **3** also displayed a similar but potent inhibition (higher that 90%) in the ATCC strain. It can be hypothesized that this compound may act in the same mechanism of biofilm formation, and that may be unaltered in both strains. It is noteworthy that compound **3** also presented inhibition of efflux pumps in *S. aureus* 272123 (Figure 2), opening the possibility of these results being related. The antibacterial sulfamide **12** could also inhibit approximately 53% of biofilm formation in this strain. 

Concerning the QS inhibition assay, the sensor strain *Chromobacterium violaceum* CV026 and the AHL producer strain *Sphingomonas paucimobilis* Ezf 10-17 (EZF) were inoculated as parallel lines, and the AHL producers *Chromobacterium violaceum* wild-type 85 (wt85) and *Serratia marcescens* AS-1 were inoculated as a single line. The interaction between the strains and compounds **2**–**18** was evaluated as the reduction of pigment production, in millimeters (mm) (Table 5) with promethazine (PMZ) as a positive control.

From the analysis of Table 5, it is noteworthy that, in general, the compounds tested were more effective against the inhibition of QS in EZF+CV026, with seven compounds (**3**, **7**, **8**, **13**, **15**, **16**, and **17**) presenting an inhibition of pigment production. Two compounds (**11** and **15**) were active against the inhibition of QS in *S. marcescens*, and no compounds were active against wt85. The potential of thioxanthone derivatives as QS inhibitors should not come as a surprise, due to their structural similarity to PMZ, which was used as a positive control in this assay [29].

### 2.7. In Vitro Studies in Mammalian Cells

#### 2.7.1. Cytotoxicity in Mouse Embryonic Fibroblast Cell Line

The cytotoxicity of the most promising derivatives, **3**, **8**, **12**, **13**, and **17**, which presented good results in the overall assays performed, such as antibacterial activity, synergy, efflux pump inhibition assay plus biofilm inhibition and/or QS inhibition assays, was investigated for toxicity using a mouse embryonic fibroblast cell line (NIH/3T3). The IC_50_ values of the tested compounds were >100 µM, except for compound **12**, with an IC_50_ of 64.47 ± 4.54 µM. Doxorubicin was used as a positive control, with an IC_50_ value of 12.05 ± 0.81 µM (Table S1, Supplementary Data).

#### 2.7.2. Compounds’ Cytotoxicity in Human Cells

**Figure 4:**
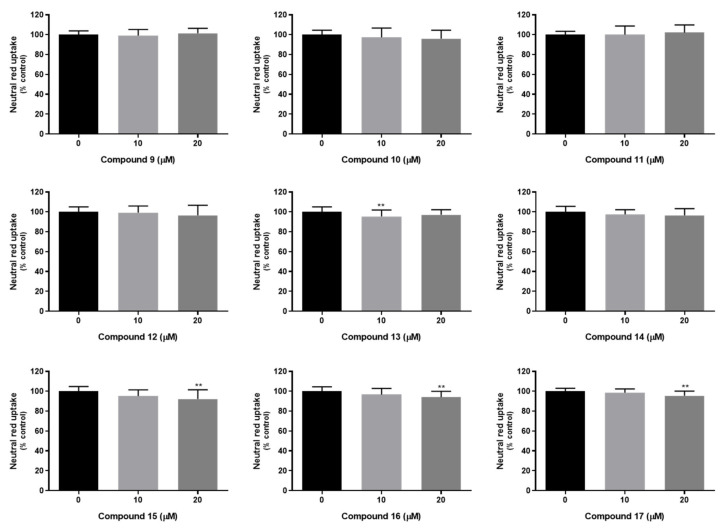
Cytotoxicity of the thioxanthone derivatives **9**–**17** (0–20 μM), in Caco-2 cells, by the neutral red (NR) uptake assay, 24 h after exposure. The results are presented as mean ± SD from four independent experiments, carried out in triplicate. Statistical comparisons were performed using the one-way ANOVA parametric method, followed by Dunnett’s multiple comparisons test [** *p* <0.01 vs. control (0 μM)].

For the newly synthetized thioxanthones **9–17** the potential for P-glycoprotein inhibition was also explored. Thus, the cytotoxicity of the thioxanthone derivatives **9–17** (0–20 μM) was initially evaluated, in Caco-2 cells, 24 h after exposure, using two different cytotoxicity assays; the neutral red (NR) uptake and the sulforhodamine B (SRB) binding assays, which are based on different principles to measure cellular dysfunction as previously studied for compounds **2**–**7**. In these studies, the tested derivatives show efficacy in the modulation of P-gp expression and/or activity at the non-cytotoxic concentration of 20 µM and after 24 h of exposure, which justifies the incubation time and concentration range used [30]. As observed in Figure 4, no significant differences in NR uptake were observed 24 h after exposure to compounds **9–12**, and **14**, at any tested concentration (10 and 20 µM), and when compared to control cells (0 µM), demonstrating the lack of lysosomal impairment. For compounds **15**, **16**, and **17**, a slight but significant decrease in the NR uptake was observed for the highest tested concentration (20 µM), and when compared to control cells (NR uptake significantly decreased to 92.2, 94.1 and 95.5%, 24 h after exposure to compounds **15**, **16**, and **17**, respectively) (Figure 3). For compound **13**, no significant effect on NR uptake was detected 24 h after exposure to the highest tested concentration (20 µM), while a slight but significant decrease in the NR uptake was observed for the 10 µM concentration (NR uptake significantly decreased to 95.5% over control cells, 24 h after exposure to 10 µM compound **13**).

**Figure 5:**
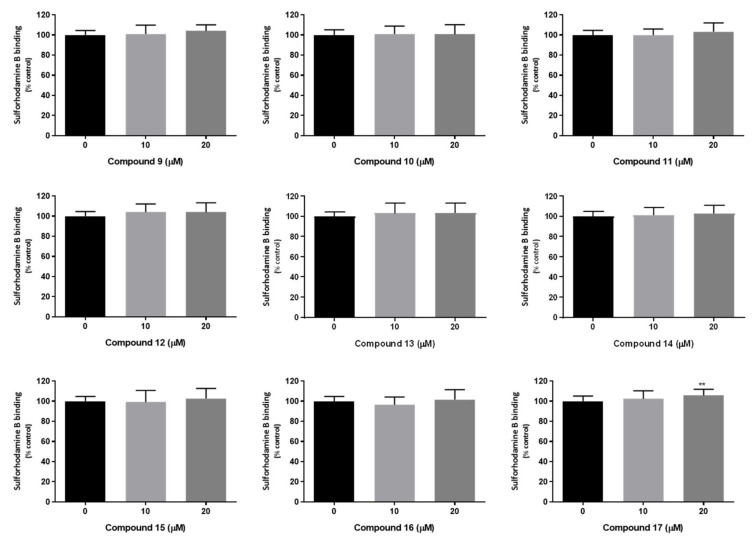
Cytotoxicity of the thioxanthone derivatives **9**–**17** (0–20 μM), in Caco-2 cells, by the sulforhodamine B binding assay, 24 h after exposure. The results are presented as mean ± SD from four independent experiments, carried out in triplicate. Statistical comparisons were performed using the one-way ANOVA parametric method, followed by Dunnett’s multiple comparisons test [** *p* <0.01 vs. control (0 μM)].

In the SRB binding assay, and as observed in Figure 5, no significant differences were detected in the ability of the dye to bind to the basic amino acids of cellular proteins, 24 h after exposure to compounds **9–16**, at any tested concentration (10 and 20 µM), demonstrating the lack of significant differences in the total protein mass and, consequently, in the number of cells in culture. In what concerns compound **17**, no significant differences in SRB binding were detected 24 h after exposure to 10 µM concentration, but a slight but significant increase in SRB binding was observed for the highest tested concentration (20 µM), and when compared to control cells (SRB binding significantly increased to 105.8% 24 h after exposure to 20 µM compound **17**).

Overall, the obtained results demonstrate the lack of relevant cytotoxicity of the thioxanthone derivatives towards Caco-2 cells, for concentrations up to 20 µM, and 24 h after exposure. Furthermore, the high agreement between the results obtained in the NR uptake and SRB binding assays should be highlighted, which provides a high confidence in the obtained data. Based on these results, the non-cytotoxic 20 µM concentration was selected for the evaluation of the potential effects of compounds in P-gp expression and activity. In fact, the ability of similar thioxanthone derivatives **2**–**7** for P-gp induction/activation has already been reported, at the same concentration and using the same in vitro model, which was the Caco-2 cells [30].

#### 2.7.3. Effects on P-gp Expression and Activity

**Figure 6:**
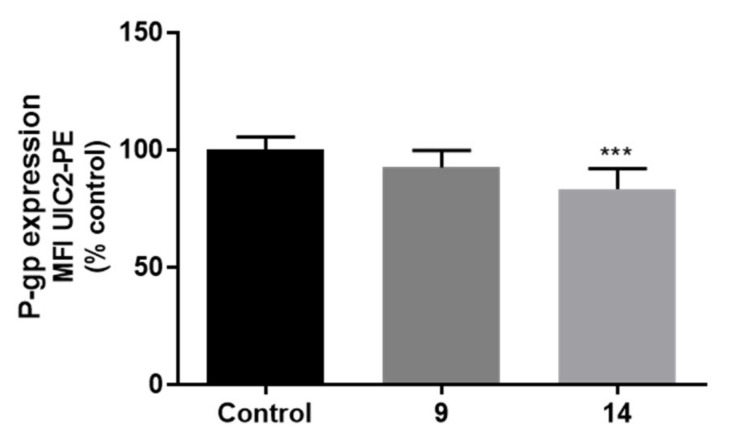
Evaluation of the expression levels of P-glycoprotein (P-gp), by flow cytometry, in Caco-2 cells exposed to compounds **9** and **14** (20 μM) for 24 h. The results are presented as mean ± SD of three independent experiments (performed in triplicate). Statistical comparisons were made using the one-way ANOVA parametric method, followed by Dunnett’s multiple comparisons test [*** *p* < 0.001 vs. control (0 μM)].

The effect of compounds (20 µM) on P-gp protein expression was evaluated, by flow cytometry, 24 h after exposure, using the monoclonal UIC2-PE antibody, as was previously studied for the other compounds **2–7**. However, the ability of compounds **9–17** to interfere with the fluorescence of the UIC2-PE antibody was assessed first. For this purpose, Caco-2 cells were incubated for 24 h, in the presence or absence of thioxanthone derivatives (20 μM), and subsequently analysed, by flow cytometry, as described for the P-gp expression assay. A remarkable increase in the intracellular fluorescence of cells exposed to compounds **10–13** and **15**–**17** was observed, making it impossible to assess the effect of these thioxanthone derivatives on P-gp expression. In fact, compounds **15–17** had previously been reported as fluorescent compounds [15] and this characteristic limited some of the studies reported herein. Only compounds **9** and **14** were tested further for their potential to modulate P-gp expression. As observed in Figure 6, no significant differences in P-gp expression in cells pre-exposed to compound **9** for 24 h were detected, when compared to control cells (0 µM). In contrast, compound **14** caused a significant reduction in P-gp expression levels (to 83%), when compared to control cells (0 µM, 100%).

**Figure 7:**
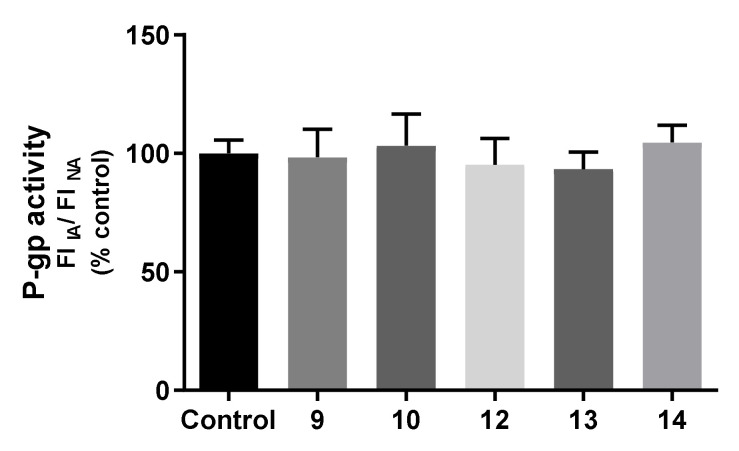
P-glycoprotein (P-gp) activity assessed by fluorescence spectroscopy in Caco-2 cells pre-exposed to the thioxanthone derivatives **9**, **10**, and **12**–**14** (20 μM) for 24 h. The results are presented as mean ± SD of five independent experiments, performed in triplicate. Statistical comparisons were made using the one-way ANOVA parametric method, followed by Dunnett’s multiple comparisons test.

The effects of the compounds (20 µM) on P-gp activity were further assessed, by fluorescence spectroscopy, using rhodamine (RHO) 123 as a P-gp fluorescent substrate and zosuquidar (ZOS) as a specific third generation P-gp inhibitor. For that purpose, two experimental designs were performed. In the first experimental approach, the accumulation of the P-gp fluorescent substrate was assessed in Caco-2 cells pre-exposed to compounds (20 μM) for 24 h, aiming to elucidate possible changes in P-gp activity resulting from changes in P-gp expression, given the long period of incubation with the thioxanthone derivatives. The combination of the evaluation of both P-gp expression and activity is extremely important, as an increased P-gp protein expression may not be necessarily accompanied by an increased activity of the pump [30,31]. Preliminary studies to assess the potential interference of compounds’ autofluorescence in the analysis of P-gp activity were also performed. Accordingly, compounds **11**, **15**, **16**, and **17** were not tested for their ability to modulate P-gp activity, given their remarkable fluorescence in the experimental conditions used to evaluate RHO 123 intracellular fluorescence. As observed in Figure 7, none of the tested compounds significantly changed the P-gp activity 24 h after exposure.

**Figure 8:**
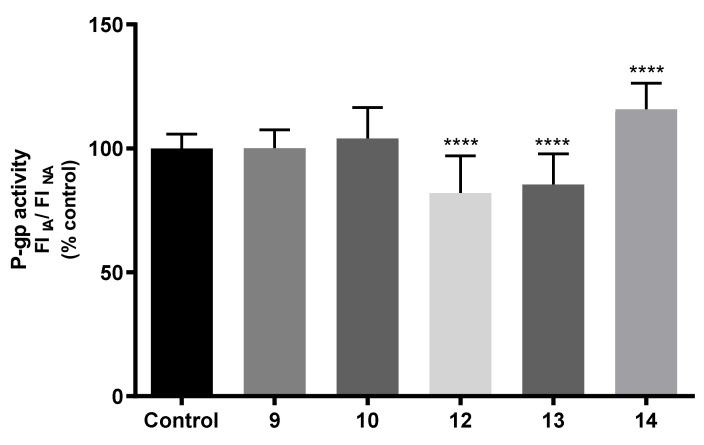
P-glycoprotein (P-gp) activity assessed by fluorescence spectroscopy in Caco-2 cells pre-exposed to thioxanthone derivatives **9**, **10**, and **12**–**14** (20 μM) only during the 90 min of the incubation period with the fluorescent substrate (RHO 123, 10 μM). The results are presented as mean ± SD of four independent experiments, performed in triplicate. Statistical comparisons were made using the one-way ANOVA parametric method, followed by Dunnett’s multiple comparisons test [**** *p* < 0.0001 vs. control (0 μM)].

Concerning the second experimental approach used to evaluate the effects of the compounds in P-gp activity, Caco-2 cells were exposed to thioxanthone derivatives (20 µM) only during the 90 min of incubation with the fluorescent substrate (RHO 123, 10 µM). This experimental approach aims to evaluate the potential immediate effects of thioxanthone derivatives on P-gp activity as a result of a direct inhibition or activation of the protein, i.e., not to change the protein expression. According to the obtained results, and as observed in Figure 7, no significant differences in P-gp activity were detected in Caco-2 cells exposed to compounds **9** and **10**, when compared to control cells (0 µM). In contrast, compounds **12** and **13** caused a significant and immediate decrease in P-gp activity (to 82 and 85%, respectively), when compared to control cells (0 µM). On the other hand, the incubation of Caco-2 cells with compound **14** resulted in a slight, but significant, immediate increase in P-gp activity (116%), when compared to control cells (100%). Therefore, compounds **12** and **13** demonstrated potential as P-gp inhibitors, while compound **14** showed itself to be an activator of this important efflux protein, overall suggesting a potential new source of new P-gp modulators. Furthermore, although compound **14** significantly reduced P-gp expression in Caco-2 cells 24 h after exposure (Figure 6), this decrease did not translate into a concomitant decrease in the protein activity (Figure 7). A possible explanation for this result could be a possible contribution of the protein activation, mediated by compound **14,** that could have remained intracellularly (after the 24-hour pre-exposure period), in agreement with its P-gp activation potential, which was observed in the experimental approach of evaluation of P-gp activity in Caco-2 cells incubated with compound **14**, only during the accumulation with the P-gp fluorescent substrate (Figure 8).

The results obtained with this assay may lead to some conclusions. Firstly, compound **12** presents both antibacterial activity and P-gp inhibition, which can pose as a limitation for its use as an antibacterial since it can compromise detoxification pathways. Compound **13**, on the other hand, presents activity regarding the inhibition of P-gp and can also inhibit the efflux systems in SE03. This may lead to the assumption that this compound is a dual inhibitor of human and bacterial efflux pumps. Further studies are needed to investigate which efflux pump is being inhibited in SE03 specifically.

## 3. Materials and Methods

All reagents and solvents used for the synthesis were purchased from Sigma-Aldrich (Sigma-Aldrich Co. Ltd., Gillinghan, UK) and no further purification process was implemented. Solvents were evaporated using a rotary evaporator under reduced pressure, Buchi Waterchath B-480. All reactions were monitored by thin-layer chromatography (TLC) carried out on precoated plates with 0.2 mm of thickness using Merck silica gel 60 (GF_254_) with appropriate mobile phases. Compounds were easily detectable at 254 nm or 365 nm.

Flash column chromatography using silica gel 60 (0.040–0.063 mm, Merck, Darmstadt, Germany) was used in the purification of the synthesized compounds. Melting points (mp) were measured in a Köfler microscope (Wagner and Munz, Munich, Germany) and are uncorrected. ^1^H- and ^13^C-nuclear magnetic resonance (NMR) spectra were recorded at the University of Aveiro, Department of Chemistry in CDCl_3_ or DMSO-d_6_ (Deutero GmbH, Ely, UK) at room temperature on a Bruker Avance 300 spectrometer (300.13 MHz for ^1^H and 75.47 MHz for ^13^C, Bruker Biosciences Corporation, Billerica, MA, USA). Chemical shifts are expressed in δ (ppm) values relative to tetramethylsilane (TMS) as an internal reference. Coupling constants are reported in Hertz (Hz). ^13^C-NMR assignments were made by bidimensional heteronuclear single quantum coherence (HSQC) and heteronuclear multiple bond correlation (HMBC) NMR experiments (long-range C, H coupling constants were optimized to 7 Hz) or by comparison with the assignments of similar molecules. High-resolution mass spectroscopy (HRMS) spectra were measured on a Bruker FTMS APEX III mass spectrometer (Bruker Corporation, Billerica, MA, USA) and recorded as electrospray ionization (ESI) mode in Centro de Apoio Cientifico e Tecnolóxico á Investigación (CACTI, University of Vigo, Pontevedra, Spain) or on a LTQ Orbitrap XL hybrid mass spectrometer (Thermo Fischer Scientific, Bremen, Germany) at CEMUP, University of Porto, Portugal. 

Quantitative and qualitative GC-MS analyses were performed on a Trace GC 2000 Series ThermoQuest gas chromatograph equipped with ion-trap GCQ Plus ThermoQuest Finnigan mass detector (Austin, TX, USA). Chromatographic separation was achieved using a capillary column (30 m 0.25 mm 0.25 µm, cross-linked 5% diphenyl and 95% dimethyl polysiloxane) from Restek^®^ (Huntingdon, PA, USA) and high-purity helium C-60 as carrier gas. An initial temperature of 80 °C was maintained for 1 minute, increased to 300 °C at 10 °C/min rate, and held for 5 min, giving a total run time of 45 min. The flow of the carrier gas was maintained at 1.5 mL/min. The injector port was set at 280 °C.

The culture media used in the experiments were the following: cation-adjusted Mueller-Hinton broth (MHB II; Sigma-Aldrich, St. Louis, MO, USA and Biokar Diagnostics, Allone, Beauvais, France), Luria-Bertani broth (LB-B; Sigma, St. Louis, MO, USA), Tryptic Soy broth (TSB; Scharlau Chemie S. A., Barcelona, Spain), and Tryptic-Soy agar (TSA; Biokar Diagnostics, Allone, Beauvais, France) were purchased. The modified Luria-Bertani agar (LB*-A), used for the quorum sensing (QS) inhibition assays, was prepared in-house, according to the formula: 1.0 g yeast extract (Merck, Darmstadt, Germany), 10.0 g tryptone (Biolab, Budapest, Hungary), 10.0 g NaCl (Molar Chemicals, Halásztelek, Hungary), 1.0 g K_2_HPO_4_ (Biolab, Budapest, Hungary), 0.3 g MgSO_4_ x 7H_2_O (Reanal, Budapest, Hungary), 5 ml Fe-EDTA stock solution and 20.0 g of bacteriological agar (Molar Chemicals, Halásztelek, Hungary) per 1 L of media.

Dulbecco’s Modified Eagle’s Medium (DMEM) with 4.5 g/L glucose, neutral red solution 0.33% (NR), Sulforhodamine B (SRB), rhodamine 123 (RHO 123), zosuquidar (ZOS), dimethylsulfoxide (DMSO) and Trizma^®^ base were obtained from Sigma (St. Louis, MO, USA). Nonessential amino acids (NEAA), heat-inactivated fetal bovine serum (FBS), antibiotic (10,000 U/mL penicillin, 10,000 μg/mL streptomycin), Hank’s Balanced Salt Solution with or without calcium and magnesium (HBSS (+/+) and HBSS (-/-), respectively) and 0.25% trypsin/1 mM ethylenediaminetetraacetic acid (EDTA) were purchased from Gibco Laboratories (Lenexa, KS, USA). Triton^TM^ X100 was purchased from Thermo Fisher Scientific (Waltham, MA, USA). Dulbecco’s Phosphate-Buffered Saline solution without calcium, magnesium and phenol red (PBS (-/-)) was purchased from Grisp (Porto, Portugal). P-gp monoclonal antibody (clone UIC2) conjugated with phycoerythrin (PE), was purchased from Abcam (Cambridge, UK). Reagents used in flow cytometry, namely, the cleaning solution, decontamination solution and flow cell extended clean solution were purchased from BD Biosciences (San Jose, CA, USA).

Dimethyl sulfoxide (DMSO), 3-(4,5-dimethylthiazol-2-yl)-2,5-diphenyltetrazolium bromide (MTT), sodium dodecyl sulfate (SDS), phosphate-buffered saline (PBS; pH 7.4), ethidium bromide (EB), reserpine, carbonyl cyanide 3-chlorophenylhydrazone (CCCP), promethazine (PMZ) and crystal violet (CV) were purchased from Sigma-Aldrich Chemie GmbH (Steinheim, Germany). Doxorubicin 2 mg/mL was purchased from Teva Pharmaceuticals, Budapest, Hungary. The antibiotic cefotaxime (CTX) was purchased from Duchefa Biochemie (Haarlem, The Netherlands) and vancomycin (VAN) from Oxoid (Basingstoke, UK). Bacteria were purchased from ATCC, the cell line NIH/3T3 was purchased from Sigma (Steinheim, Germany) and Caco-2 cells were obtained from the European Collection of Cell Culture (ECACC, Salisbury, UK).

### 3.1. Chemistry

The syntheses of compounds **2**–**7** [11] and **15**–**18** [15] have been previously reported. Compounds **8–13** were synthesized in the same way as **15–18**, through a copper-catalyzed Ullmann-type C–N coupling, starting from compound **1** or **14**. The general procedures were as follows.

#### 3.1.1. General Procedure for the Synthesis of 1-Nitrogen Substituted Thioxanthones (8–13) 

Compounds **8**–**13** were obtained by adding the appropriate amine or sulfamide to a suspension of 1-chloro-4-propoxy-9*H*-thioxanthen-9-one (**1**, 0.250 mg, 0.8 mmol) (procedure **a**) or 1-bromo-4-propoxy-9*H*-thioxanthen-9-one (**14**, 0.250 mg, 0.7 mmol) (procedure **b**) in methanol (25 mL), CuI (2 mg) and K_2_CO_3_ (0.1 mmol). The suspension was heated at 100 °C for 48 h, in a sealed flask.

1-[(4,6-Dimethylpyrimidin-2-yl)amino]-4-propoxy-9*H*-thioxanthen-9-one (**8**): 3% as red dust (procedure **a**). mp 179.7–181.7 °C (methanol). IR (KBr)_max_: 3445, 2966, 2919, 2878, 2851, 1594, 1575, 1505, 1475, 1465, 1443, 1401, 1384, 1335, 1291, 1267, 1240, 1226, 1166, 1116, 1086, 1063, 973, 890, 822, 789, 769, 629. ^1^H NMR (CDCl_3_, 300.13 MHz) δ (ppm): 12.8 (1H, s, NH), 9.01 (1H, d, J = 9.21, H2), 8.62 (1H, dd, J = 8.13 and 1.94 Hz, H8), 7.58 (2H, m, H5 and H6), 7.45 (1H, ddd, J = 8.19, 5.10 and 3.14 Hz, H7), 7.22 (1H, d, J = 9.24 Hz, H3), 6.53 (1H, s, H3′), 4.12 (2H, t, J = 6.44 Hz, Ha), 2.42 (6H, s, H5′ and H6′), 1.93 (2H, st, J = 7.22 Hz, Hb), 1.14 (3H, t, J = 7.41 Hz, Hc). ^13^C NMR (CDCl_3_, 75.48 MHz) δ (ppm): 184.0 (C9), 167.5 (C2′ and C4′), 159.9 (C1′), 146.7 (C4), 139.9 (C1), 136.9 (C10a), 132.1 (C6), 130.2 (C8a), 129.9 (C8), 129.0 (C4a), 126.3 (C7), 126.0 (C5), 116.6 (C3), 116.4 (C9a), 115.6 (C2), 112.2 (C3′), 71.8 (Ca), 24.2 (C5′ and C6′), 22.8 (Cb), 10.9 (Cc) (Figure S13, Supplementary Data).

4-(9-Oxo-4-propoxy-9*H*-thioxanthen-1-yl)piperazin-2-one (**9**): 12% as red dust (procedure **a**). mp >350 °C (methanol). IR (KBr)_max_: 3435, 2963, 2922, 2852, 2360, 1604, 1508, 1457, 1430, 1395, 1348, 1320, 1291, 1261, 1232, 1170, 1106, 1061, 998, 967, 919, 809, 744, 732, 621, 575, 529, 472, 418. ^1^H NMR (DMSO, 500.13 MHz) δ (ppm): 8.55 (1H, s, NH), 7.90 (1H, dd, J = 7.70 and 1.38 Hz, H8), 7.51 (1H, dd, J = 7.80 and 1.09 Hz, H5), 7.40 (1H, td, J = 7.98 and 1.57 Hz, H6), 7.35 (1H, td, J = 7.45 and 1.27 Hz, H7), 7.00 (1H, d, J = 9.00 Hz, H3), 6.91 (1H, d, J = 8.90 Hz, H2), 3.98 (2H, t, J = 6.38 Hz, Ha), 3.86 (2H, t, J = 5.30 Hz, H1′), 3.73 (2H, t, J = 5.53 Hz, H2′), 3.29 (2H, s, H4′), 1.75 (2H, st, J = 7.32 Hz, Hb), 1.03 (3H, t, J = 7.40 Hz, Hc). ^13^C NMR (DMSO, 75.48 MHz) δ (ppm): 171.8 (C3′), 163.1 (C9), 146.6 (C4), 144.9 (C1), 134.9 (C8a), 131.7 (C10a), 128.8 (C6), 126.8 (C8), 126.3 (C7), 125.2 (C5), 122.5 (C4a), 121.0 (C9a), 117.3 (C2), 114.2 (C3), 70.3 (Ca), 60.7 (C1′), 50.2 (C2′), 57.7 (C4′), 22.3 (Cb), 10.6 (Cc) (Figure S14, Supplementary Data). HRMS (ESI+): m/z [C_20_H_20_N_2_O_3_S + H]^+^ calcd. for [C_20_H_21_N_2_O_3_S]: 369.1273; found 369.1263 (Figure S15, Supplementary Data).

1-[(2-Hydroxyethyl)amino]-4-propoxy-9*H*-thioxanthen-9-one (**10**): 7% as red needles (procedure **a**). mp 173.1–173.9 °C (methanol). IR (KBr)_max_: 3447, 2360, 2342, 1636, 669, 649, 494, 458. ^1^H NMR (CDCl_3_, 300.13 MHz) δ (ppm): 10.1 (1H, s, OH), 8.49 (1H, td, *J* = 8.1 and 1.1 Hz, H8), 7.53 (2H, m, H5 and H7), 7.40 (1H, ddd, *J* = 8.2, 4.6 and 3.7 Hz, H6), 7.12 (1H, d, *J* = 9.0 Hz, H3), 6.61 (1H, d, *J* = 9.1 Hz, H2), 4.01 (2H, t, *J* = 6.5 Hz, Ha), 3.96 (2H, t, *J* = 5.4 Hz, H2′), 3.46 (2H, t, *J* = 5.4 Hz, H1′), 1.88 (2H, st, *J* = 7.4 Hz, Hb), 1.12 (3H, t, *J* = 7.4 Hz, Hc). ^13^C NMR (CDCl_3_, 125.77 MHz) δ (ppm): 183.6 (C9), 148.5 (C1), 143.1 (C4), 136.9 (C10a), 132.6 (C5), 130.2 (C9a), 129.8 (C8a), 129. 3 (C8), 126.0 (C6 and C7), 119.3 (C3), 112.8 (C4a), 107.8 (C2), 72.7 (Ca), 61.3 (C2′), 45.9 (C1′), 23.0 (Cb), 10.9 (Cc) (Figure S16, Supplementary Data). HRMS (ESI+): m/z calcd. for [C_18_H_19_NO_3_S]: 329.1086; found 329.1066 (Figure S17, Supplementary Data).

1-[(2-Morpholinoethyl)amino]-4-propoxy-9*H*-thioxanthen-9-one (**11**): 45% as orange needles (procedure **a**). mp 126.4–127.7 °C (methanol). IR (KBr)_max_: 3448, 3287, 2963, 2924, 2853, 2818, 2363, 1739, 1615, 1574, 1497, 1466, 1443, 1435, 1392, 1294, 1270, 1245, 1226, 1171, 1147, 1115, 1072, 1037, 794, 746, 722. ^1^H NMR (CDCl_3_, 500.13 MHz) δ (ppm): 9.90 (1H, t, J = 4.50 Hz, NH), 8.52 (1H, dd, J = 8.01 and 1.86 Hz, H8), 7.52 (2H, m, H5 and H6), 7.40 (1H, ddd, J = 8.16, 4.79 and 3.44 Hz, H7), 7.13 (1H, d, J = 9.06 Hz, H3), 6.52 (1H, d, J = 9.06 Hz, H2), 4.01 (2H, t, J = 6.50 Hz, Ha), 3.79 (4H, t, J = 4.65 Hz, H3′ and H4′), 3.36 (2H, q, J = 6.51 Hz, H1′), 2.76 (2H, t, J = 6.59 Hz, H2′), 2.57 (4H, t, J = 4.55 Hz, H3′ and H6′), 1.88 (2H, st, J = 7.32 Hz, Hb), 1.11 (3H, t, J = 7.40 Hz, Hc). ^13^C NMR (CDCl_3_, 75.48 MHz) δ (ppm): 183.4 (C9), 149.2 (C1), 142.6 (C4), 136.8 (C10a), 131.7 (C6), 130.4 (C8a), 129.8 (C4a), 129.3 (C8), 126.0 (C5), 125.9 (C7), 120.3 (C3), 113.2 (C10a), 106.5 (C2), 72.8 (Ca), 67.2 (C4′ and C5′), 57.2 (C2′), 53.8 (C3′ and C6′), 40.6 (C1′), 23.1 (Cb), 10.7 (Cc) (Figure S18, Supplementary Data). HRMS (ESI+): m/z [C_22_H_26_N_2_O_3_S + H]^+^ calcd. for [C_22_H_27_N_2_O_3_S]: 399.1742; found 399.1749 (Figure S19, Supplementary Data).

4-[*N*-(9-Oxo-4-propoxy-9*H*-thioxanthen-1-yl)sulfamoyl]benzoic acid (**12**): 3% (procedure **a**) and 48% as orange powder (procedure **b**). mp 324.6–326.4 °C (chloroform). IR (KBr)_max_: 3440, 2967, 2879, 1595, 1554, 1460, 1437, 1383, 1357, 1292, 1266, 1205, 1165, 1133, 1087, 1061, 992, 832, 787, 751, 729, 699, 628, 604, 557. ^1^H NMR (DMSO, 300.13 MHz) δ (ppm): 12.7 (1H, s, OH), 8.44 (1H, dd, *J* = 8.28 and 1.44, H8), 7.88 (3H, d, *J* = 8.07 Hz, H5, H3′ and H5′), 7.77 (1H, ddd, *J* = 8.22, 6.92 and 1.38 Hz, H6), 7.71 (2H, d, *J* = 8.01, H2′ and H6′), 7.59 (1H, ddd, *J* = 8.25, 6.95 and 1.38 Hz, H7), 7.51 (1H, d, *J* = 8.94 Hz, H2), 7.43 (1H, d, *J* = 8.94 Hz, H3), 4.10 (2H, t, *J* = 6.30 Hz, Ha), 1.78 (2H, st, *J* = 7.04 Hz, Hb), 1.02 (3H, t, *J* = 7.38 Hz, Hc). ^13^C NMR (DMSO, 75.48 MHz) δ (ppm): 185.2 (C9), 168.1 (C7′), 149.2 (C4), 145.2 (C4′), 138,0 (C1′), 136.1 (C10a), 134.2 (C1), 133.5 (C6), 129.6 (C3′ and C5′), 129.1 (C8), 128.8 (C4a), 128.0 (C9a), 127.2 (C7 and C8a), 126.8 (C5), 126.2 (C2′ and C6′), 115.9 (C2 and C3), 72.3 (Ca), 22.7 (Cb), 10.5 (Cc) (Figure S20, Supplementary Data). HRMS (ESI+): m/z [C_23_H_19_NO_6_S_2_ + H]^+^ calcd. for [C_23_H_20_NO_6_S_2_]: 470.0732; found 470.0726 (Figure S21, Supplementary Data).

4-Amino-*N*-(9-oxo-4-propoxy-9*H*-thioxanthen-1-yl)benzenesulfonamide (**13**): 28% as orange crystals (procedure **b**). mp 211.9–213.5 °C [dichloromethane (9):acetone (1)]. IR (KBr)_max_: 3479, 3377, 2968, 2879, 1630, 1591, 1569, 1500, 1460, 1435, 1362, 1316, 1287, 1264, 1211, 1185, 1176, 1147, 1089, 1061, 1008, 984, 939, 829, 745, 722, 673, 659, 635, 563, 547, 527, 474, 459. ^1^H NMR (CDCl_3_, 300.13 MHz) δ (ppm): 12.4 (1H, s, NH), 8.51 (1H, dd, *J* = 8.18 and 1.97 Hz, H8), 7.66 (1H, d, *J* = 8.94 Hz, H3), 7.60 (4H, m, H5, H6, H2′ and H6′), 7.47 (1H, ddd, *J* = 8.22, 6.05 and 2.21 Hz, H7), 7.06 (1H, d, *J* = 9.00 Hz, H2), 6.51 (2H, dt, *J* = 9.24 and 2.36 Hz, H3′ and H5′), 4.07 (2H, t, *J* = 6.36 Hz, Ha), 1.90 (2H, st, *J* = 7.37 Hz, Hb), 1.12 (3H, t, *J* = 7.41 Hz, Hc). ^13^C NMR (CDCl_3_, 125.77 MHz) δ (ppm): 184.2 (C9), 150.2 (C1), 149.2 (C4), 137.3 (C4′), 137.2 (C10a), 136.2 (C8a and C9a), 132.7 (C6), 129.8 (C8), 129.6 (C2′ and C6′), 129.3 (C1′), 126.6 (C7), 126.4 (C4), 117.9 (C4a), 116.6 (C3), 115.0 (C2) 114.2 (C3′ and C5′), 71.5 (Ca), 22.7 (Cb), 10.8 (Cc) (Figure S22, Supplementary Data). HRMS (ESI+): m/z [C_22_H_20_N_2_O_4_S_2_ + Na]^+^ calcd. for [C_23_H_19_NO_6_S_2_Na]: 462.0683; found 462.2658 (Figure S23, Supplementary Data).

#### 3.1.2. General Procedure for the Synthesis of 1-Bromo-4-Propoxy-9*H*-Tioxanthen-9-One (14)

From a suspension of 1-chloro-4-propoxy-9*H*-tioxanthen-9-one (**1**) (400 mg, 1 mmol) in nitrobenzene (10 mL), KBr (5 mmol), CuI (0.01 mmol) and H_3_PO_4_ (8 mmol) (procedure **c**), compound **14**, a bromine derivative, was obtained. The reaction mixture stirred at 200 °C (reflux) for 48 h.

1-Bromo-4-propoxy-9*H*-thioxanthen-9-one (**14**): 54% as brown needles (procedure **c**); mp 88-90 °C (methanol). IR (KBr)_max_: 3442, 2961, 2874, 2360, 1637, 1592, 1580, 1560, 1460, 1427, 1375, 1307, 1254, 1173, 1114, 1053, 961, 911, 865, 819, 807, 787, 753, 745, 677, 642, 566, 478. ^1^H NMR (CDCl_3_, 300.13 MHz) δ (ppm): 8.45 (1H, dd, J = 8.01 and 0.83 Hz, H8), 7.66 (1H, d, J = 8.61 Hz, H3), 7.58 (2H, m, H5 and H6), 7.44 (1H, ddd, J = 8.13, 5.40 and 2.80 Hz, H7), 6.89 (1H, d, J = 8.61 Hz, H2), 4.11 (2H, t, J = 6.39 Hz, Ha), 1.94 (2H, st, J = 7.35 Hz, Hb), 1.14 (3H, t, J = 7.41 Hz, Hc). ^13^C NMR (CDCl_3_, 75.48 MHz) δ (ppm): 180.5 (C9), 153.3 (C4), 135.7 (C8a), 133.2 (C3), 132.1 (C6), 130.6 (C1), 130.5 (C9a), 129.8 (C8), 127.4 (C10a), 126.7 (C7), 126.1 (C5), 113.7 (C4a), 113.2 (C2), 71.4 (Ca), 22.6 (Cb), 10.8 (Cc) (Figure S24, Supplementary Data). MS (EI) *m/z* (%): 349.9 (9) [M + 2]^+.^, 347.9 (10) [M]^+.^, 307.9 (32), 305.9 (32), 279.9 (20), 277.9 (20), 227.0 (65), 199.0 (57), 171.0 (100) (Figure S25, Supplementary Data).

### 3.2. X-ray Crystallography 

Diffraction data were collected at 291 K with a Gemini PX Ultra (Rigaku/Oxford, Neu-Isenburg, Germany) equipped with CuKα radiation (λ = 1.54184 Å). The structures were solved by direct methods using SHELXS-97 [32] and refined with SHELXL-97 [32]. Carbon, oxygen, nitrogen, and sulfur atoms were refined anisotropically. Hydrogen atoms were either placed at their idealized positions using appropriate HFIX instructions in SHELXL, and included in subsequent refinement cycles, or were directly found from difference Fourier maps and were refined freely with isotropic displacement parameters. Full details of the data collection and refinement and tables of atomic coordinates, bond lengths and angles, and torsion angles have been deposited with the Cambridge Crystallographic Data Centre (CCDC).

4-Amino-*N*-(9-oxo-4-propoxy-9*H*-thioxanthen-1-yl)benzenesulfonamide (**13**): Crystal was monoclinic, space group P2_1_/n, cell volume 2018.0(11) Å^3^ and unit cell dimensions a = 11.016(3) Å, b = 8.542(4) Å and c = 21.450(4) Å, and β = 91.252(17)° (uncertainties in parentheses). The refinement converged to R (all data) = 7.92% and wR2 (all data) = 17.94%. CCDC 2081536.

1-Bromo-4-propoxy-9*H*-thioxanthen-9-one (**14**): Crystal was triclinic, space group P-1, cell volume 2018.0(11) Å^3^ and unit cell dimensions a = 11.3197(8) Å, b = 11.9631(8) Å and c = 12.7932(10) Å, and cell angles α = 108.678(6)°, β = 111.986(7)° and γ = 99.290(6)° (uncertainties in parentheses). The refinement converged to R (all data) = 11.96% and wR2 (all data) = 26.62%. CCDC 2081537.

### 3.3. Docking Studies 

The crystal structure of the AcrB (PDB: 4DX5) [33], AcrA (PDB: 2F1M) [34], and TolC (PDB: 1EK9) [35] portions of the AcrAB-TolC bacterial efflux system, downloaded from the protein databank (PDB) [36], were used for this study. The known AcrAB-TolC inhibitors D13-9001, doxorubicin, MBX-3132, minocycline, phenyl-arginyl-β-naphthylamide, reserpine, *trans*(*E*)-flupentixol, and verapamil, along with the tested compounds, were drawn with ChemDraw (PerkinElmer Informatics) and minimized using ArgusLab. Docking was carried out using AutoDock Vina (Scripps, CA, USA) [37], in the sites described in [19,21]. The NorA efflux pump does not have an available crystal structure, and a homology model was prepared. The model was generated using the Swiss Model server [38] and the sequence deposited in Uniprot (Q5HHX4) [39], using the EmrD pump from *E. coli* (PDB: 2GFP) as the homolog, as described in [22].

Regarding the P-glycoprotein (P-gp) efflux pump, the 3D structure of the ATP-bound P-gp in the outward-facing conformation, which has a low affinity for substrates and modulators, was established by cryo-electron microscopy [40]. Although the human P-gp structure in the inward-facing conformation, which has higher affinity for substrates and modulators, is still not described, there are several mice P-gp structures obtained by X-ray structures [41] and by electron density maps already published [42,43]. Therefore, a human P-gp homology model was built, using as a template the mice 3D P-gp with the highest identity percentage and the availability of 3D coordinates with the best resolution, which was 4Q9H mice P-gp, with an identity percentage of 89.0%, and a resolution of 3.4 Å [43]. After single sequence alignment, model creation, incorporation of missing loops to the model, insertion of side chains, verification of overall geometry, addition of hydrogen atoms, adding of charges, and final staged minimization performed using Sybyl (Tripos Certara, MO, USA), the human P-gp model was obtained and validated with a single-point energy calculation and Ramachandran plot analysis (98.7%, i.e., 1179/1194 of all residues were in the allowed regions). The top nine poses were collected for each molecule and the lowest docking score value was associated with the most favorable binding conformation. PyMOL (Schrödinger, NY, USA) [44] was used for visual inspection of results and graphical representations.

### 3.4. Bacterial Strains

As Gram-positive strains, *Staphylococcus aureus* American Type Culture Collection (ATCC) 25923, *Enterococcus faecalis* ATCC 29212, methicillin and ofloxacin-resistant *Staphylococcus aureus* 272123 clinical isolate, and environmental isolate vancomycin-resistant enterococci (VRE) *E. faecalis* B3/101 [23] were used. As Gram-negative strains, *Escherichia coli* ATCC 25922, *Pseudomonas aeruginosa* ATCC 27853, the *acrA* gene inactivated mutant *Salmonella enterica* serovar Typhimurium SL1344 (SE03), and clinical isolates of the extended-spectrum β-lactamase producer (ESBL) *E. coli* SA/2 were investigated in this study.

For the QS tests, all the strains used were Gram-negative. The bacteria used were *Chromobacterium violaceum* wild type 85 (wt85) characterized by the acyl-homoserine-lactones (AHL) signal molecule-mediated production of the purple violacein pigment, capable of endogenous QS-signal molecule-production (*N*-hexanoyl-l-HSL), *C. violaceum* CV026 (CV026), a Tn5 transposase-mutant, AHL-signal molecule indicator strain (produces purple violacein pigment in the presence of AHL), which is incapable of endogenous QS-signal molecule-production, but is useful in the detection of external stimuli, *Sphingomonas paucimobilis* Ezf 10-17 (EZF), AHL-producing-strain (used with *C. violaceum* CV026), and Serratia marcescens AS-1, characterized by the production AHL signal molecule-mediated production of the orange-red pigment prodigiosin (2-methyl-3-pentyl-6-methoxyprodigiosin), capable of endogenous QS-signal molecule-production (*N*-hexanoyl- l-HSL) [45].

### 3.5. Antibacterial Assay and Synergy with Antimicrobials

The minimum inhibitory concentration (MIC) for each compound was determined by broth microdilution method in a 96-well plate according to the Clinical and Laboratory Standard Institute (CLSI) guidelines [46]. The medium used was MHB II. The concentrations tested ranged from 100 µM to 0.195 µM and were prepared from a stock solution of 10 mM in DMSO, for *S. aureus* 272123 and SE03; for the other strains, the concentrations tested ranged from 64 µg/mL to 4 µg/mL and were prepared from a stock solution of 10 mg/mL in DMSO. DMSO was used in subinhibitory concentrations (1% *v*/*v*). The MIC was determined by visual inspection.

The combined effect of the compounds and clinically relevant antimicrobial drugs was evaluated by determining the antibiotic’s MIC in the presence of each compound. Briefly, the MIC of cefotaxime (CTX) (Duchefa Biochemie, Haarlem, The Netherlands) and vancomycin (VAN) (Oxoid, Basingstoke, UK) for *E. coli* SA/2 and *E. faecalis* B3/101, respectively, were determined in the presence of the highest concentration of each compound tested that did not affect bacterial growth when the compound was used alone. The antibiotic tested was serially diluted, whereas the concentration of each compound was kept fixed. In the case of compound **12**, the concentration used was 8 µg/mL. For all other compounds, the concentration used was 64 µg/mL. Antibiotic MICs were determined as described above.

### 3.6. Real-Time EB Accumulation Assay

The compounds were evaluated for their ability to inhibit the efflux of EB, an efflux pump substrate, in *Salmonella enterica* serovar Typhimurium SE03 and *S. aureus* 272123 strains, through real-time fluorimetry, monitoring the intracellular accumulation of EB, an efflux pump substrate. This was determined by the automated method using a CLARIOstar Plus plate reader (BMG Labtech, Ortenberg, Germany). Reserpine and CCCP were applied at 25 µM as positive controls, and the solvent DMSO was applied at 1% *v*/*v*. The bacterial strains were incubated in an appropriate culture media (TSB–*S. aureus* 272123; LB-B–SE03) at 37 °C until they reached an optical density (OD) of 0.6 at 600 nm. The culture was centrifuged at 13,000× g for 3 min, and the pellet was washed and resuspended with phosphate buffered saline (PBS, pH 7.4). The suspension was centrifuged again in the same conditions and resuspended in PBS.

Compound **12** was applied at a one-third MIC concentration, 17 µM, and the rest was applied at 50 µM, in a solution of a non-toxic concentration of EB (1 µg/mL) in PBS. Then, 50 µL of this solution were transferred into a 96-well black microtiter plate (Greiner Bio-One Hungary Kft, Mosonmagyaróvár, Fertősor, Hungary), and 50 µL of bacterial suspension (OD_600_ = 0.6) were added to each well. The plates were placed into the CLARIOstar plate reader, and the fluorescence was monitored at excitation and emission wavelengths of 530 nm and 600 nm, respectively, every minute for one hour on a real-time basis. From the real-time data, the activity of the compounds, namely the relative fluorescence index (RFI) of the last time point (minute 60) of the EB accumulation assay, was calculated according to the following equation:

(1) $$RFI=\frac{RF_{treated}-RF_{untreated}}{RF_{untreated}}$$

where RF_treated_ is the relative fluorescence (RF) at the last time point of EB accumulation curve in the presence of the compound, and RF_untreated_ is the RF at the last time point of the EB accumulation curve of the untreated control, having only the solvent (DMSO) control [13]. The accumulation curves were designed using Microsoft Excel^®^. The samples were tested in triplicate, and the RFI presented comes from the average of these three values. The accumulation graphs present the mean of the RF at the last time point. The standard deviation (SD) was calculated automatically and included in the RF graphs. 

### 3.7. Inhibition of Biofilm Formation

The thioxanthone derivatives were tested for their ability to decrease the formation of biofilm. The bacterial strains used were the Gram-positive *S. aureus* ATCC 25923 and *S. aureus* 272123. The detection of the biofilm formation was carried out using the dye crystal violet (CV; 0.1% *v*/*v*). The initial inoculum was incubated in TSB overnight, and then diluted to an OD_600_ of 0.1. Then, the bacterial suspension was added to 96-well microtiter plates and the compounds were added at a sub-MIC concentration. This meant that compound **12** was added at 50 µM, while the rest of the compounds were added at the concentration of 100 µM. The final volume in each well was 200 µL. Reserpine was used as a positive control, as it was the same compound used in the efflux pump inhibition assay and it has shown activity in the inhibition of biofilm formation in *S. aureus* strains [27]. The plates were incubated at 30 °C for 48 h, with gentle stirring (100 rpm). After this incubation period, the TSB medium was discarded, and the plates were washed with tap water to remove unattached cells. Afterwards, 200 µL of a 0.1% *v*/*v* CV solution were added to the wells and incubated for 15 min at room temperature. Then, the CV solution was removed from the wells, and the plates were washed again with tap water, and 200 µL of a 70% ethanol solution was added to the wells. The biofilm formation was determined by measuring the OD_600_ using a Multiscan EX ELISA plate reader (Thermo Labsystems, Cheshire, WA, USA). The anti-biofilm effect of the compounds was expressed in the percentage (%) of decrease of biofilm formation.

### 3.8. Quorum Sensing Assay

The QS inhibitory effect of the compounds was examined on the EZF and the sensor CV026 strains, on the wt85 strain, and on *S. marcescens*. The method used was the parallel inoculation method, where pair combinations of the used sensor strain CV026 and the *N*-acyl-homoserine lactone (AHL) producing strain EZF were inoculated directly onto the LB*-A agar surface in parallel, at an approximate distance of 5 mm from each other. *S. marcescens* AS-1 and wt85 were inoculated as a single line. Filter paper disks (7 mm in diameter) were placed on the center of the inoculated line(s) and impregnated with 8 µL of a solution of 10 mM of the compounds. PMZ was used as positive control, as previous results have demonstrated its activity as a QS inhibitor [29]. The agar plates were incubated at room temperature (20 °C) for 24−48 h. The QS inhibition was accessed visually, through the inhibition of pigment production. The discolored, but intact, bacterial colonies were measured with a ruler [29].

### 3.9. In Vitro Studies in Mammalian Cells

#### 3.9.1. Cytotoxicity in NIH/3T3 Cell Line

A 10.0 mM stock solution of each compound was prepared in DMSO. All stock solutions were stored at −80 °C and freshly diluted on the day of the experiment in a fresh cell culture medium (ensuring that DMSO did not exceed 0.1% DMSO concentration of the exposure media).

Mouse fibroblasts (NIH/3T3, ATCC CRL-1658TM) were cultivated in DMEM (Gibco 52100-039) and supplemented with 10% heat-inactivated fetal bovine serum (Biowest, VWR International Kft, Debrecen, Hungary), 2 mM of l-glutamine, 1 mM Na pyruvate, 100 U/L and 10 mg/L penicillin/streptomycin mixture (Sigma-Aldrich Chemie GmbH, Steinheim, Germany), respectively, and 0.1% nystatin (8.3 g/L in ethylene glycol). The adherent cells were detached using a combination of 0.25% Trypsin-Versene (EDTA) solution for 5 min at 37 °C. Before each cytotoxicity assay using this cell line, cells were seeded in untreated 96-well flat-bottom microtiter plates, following a 4-hour incubation period in a humidified atmosphere (5% CO_2_, 95% air) at 37 °C [47].

The cytotoxicity of **3**, **11**, **12**, **13**, and **17** was assessed in NIH/3T3 cells, using the MTT assay. Prior to the assay, the cells were seeded for 4 h using 1 × 10^4^ cells/well. The compounds were added by 2-fold serial dilutions to the cells distributed into 96-well flat bottom microtiter plates starting with 100 μM. The plates were incubated for 24 h, after which a solution of MTT in PBS was added to each well and incubated for another 4 h. After this, 100 μL of SDS (10% in a 0.01 M HCl solution) was added to each well and incubated overnight at 37 °C. Doxorubicin was used as a positive control. Cell growth was determined in quadruplicate by measuring OD at λ = 540 nm (reference 630 nm) in a Multiscan EX ELISA reader (Thermo Labsystems, Cheshire, WA, USA). The percentage of inhibition of cell growth was determined according to the Equation (2).

(2) $$100-\left(\frac{OD_{sample}-OD_{medium\;control}}{OD_{cell\;control}-OD_{medium\;control}}\right)\times 100$$

#### 3.9.2. Cytotoxicity in Caco-2 Cell Line

A 20.0 mM stock solution of each compound was prepared in DMSO. All stock solutions were stored at −80 °C and freshly diluted on the day of the experiment in a fresh cell culture medium (ensuring that DMSO did not exceed 0.1% DMSO concentration of the exposure media).

Caco-2 cells were routinely cultured in 75 cm^2^ flasks using DMEM with 4.5 g/L glucose supplemented with 10% FBS, 1% NEAA and 1% antibiotic. Cells were maintained at 37 °C, in a 5% CO_2_ and 95% air atmosphere, and the cell culture medium was changed every 2−3 days. Cultures were passaged weekly by trypsinization (0.25% trypsin/1 mM EDTA). In all experiments, the cells were seeded at a density of 60,000 cells/cm^2^ and used three days after seeding, when confluence was reached. The cells used in all experiments were taken between the 55th and 64th passages.

The compounds’ (0−20 μM) cytotoxicity was evaluated by the neutral red (NR) uptake and Sulforhodamine B (SRB) binding assays, 24 h after exposure. Caco-2 cells were seeded onto 96-well plates at a density of 60,000 cells/cm^2^ and exposed, after reaching confluence (three days after seeding), to the compounds (0–20 μM), in a fresh cell culture medium. Triton™ X-100 (0.1%) was used as a positive control.

#### 3.9.3. Neutral Red Uptake Assay

The NR assay is based on the ability of viable cells to incorporate and bind the supravital dye NR into the lysosomes, thus providing a quantitative estimation of the number of viable cells in a culture [48]. Twenty-four hours after exposure to the compounds, the cell culture medium was removed, followed by the addition of fresh cell culture medium containing 50 μg/mL NR, and incubation at 37 °C in a humidified 5% CO_2_ and 95% air atmosphere, for 90 min. After this incubation period, the cell culture medium was removed, the dye absorbed only by viable cells extracted with absolute ethyl alcohol/distilled water (1:1) with 5% acetic acid, and the absorbance measured at 540 nm in a multiwell plate reader (PowerWaveX BioTek Instruments, VT, USA). The percentage of NR uptake relatively to that of the control cells (0 µM) was used as the cytotoxicity measure. Five independent experiments were performed, in triplicate.

#### 3.9.4. Sulforhodamine B Binding Assay 

The SRB binding assay is based on the pH-dependent binding of SRB to basic amino acids of cellular proteins under mild acidic conditions. The colorimetric evaluation of bounded SRB thus provides an estimation of the total protein mass, which is related to the number of cells in culture [49]. Twenty-four hours after exposure to the compounds, the cell culture medium was removed, the cells were washed with HBSS (+/+) and fixed overnight, at −20 °C, with a methanolic solution of 1% acetic acid (*v*/*v*). The fixation medium was then removed, and the cells incubated with a 0.05% SRB solution (prepared in 1% acetic acid) for 60 min, at 37 °C. After incubation, the SRB solution was removed and the cells washed with 1% acetic acid (*v*/*v*) to remove the unbound dye. The bounded SRB was then extracted with a Tris base solution (10 mM, pH 10.5) and the absorbance was measured, at 540 nm, in a multi-well plate reader (PowerWaveX BioTek Instruments, VT, USA). The percentage of SRB binding relatively to that of the control cells (0 µM) was used as the cytotoxicity measure. Six independent experiments were performed, in triplicate.

#### 3.9.5. Flow Cytometry Analysis of P-Glycoprotein Expression

The effect of the compounds on P-gp expression was evaluated by flow cytometry, 24 h after exposure, using a P-gp monoclonal antibody (UIC2 clone conjugated with PE), as previously described [50]. Briefly, Caco-2 cells were seeded in 24-well plates at a density of 60,000 cells/cm^2^ and exposed, three days after seeding, to a non-cytotoxic concentration of the tested compounds (20 µM). After 24 h of exposure, the cell culture medium was removed, the cells washed with HBSS (-/-) and harvested by trypsinization (0.25% trypsin/1 mM EDTA) to obtain a cell suspension. The obtained cell suspension was transferred into Eppendorf tubes, centrifuged (300 g for 5 min, at 4 °C) and re-suspended in HBSS (+/+) buffer containing the P-gp antibody (the antibody dilution was applied according to the manufacturer’s instructions for flow cytometry). The incubation with the P-gp antibody was performed at 37 °C, in the dark and under gentle shaking, for 60 min. The cells were then washed twice with HBSS (+/+), centrifuged (300 g for 5 min, at 4 °C) and kept on ice until analysis. Cells were then re-suspended on ice-cold PBS (-/-) buffer immediately before the cytometer analysis, which was performed using a Becton a BD Accuri^TM^ C6 flow cytometer (BD Biosciences, CA, USA), equipped with the FCS Express analysis software. The fluorescence of the P-gp antibody (UIC2-PE) was measured by a 585 ± 40 nm band-pass filter (FL2). Logarithmic fluorescence was recorded and displayed as a single parameter histogram and based on the acquisition of data for at least 20,000 cells. The parameter used for comparison was the mean fluorescence intensity (MFI), calculated as a percentage of control cells (0 μM). The cells autofluorescence (unlabelled cells with or without exposure to the tested compounds) was also evaluated to eliminate its potential contribution to the analysed fluorescence signals. Given the high autofluorescence of several of the tested compounds, only compounds **9** and **14** were able to be tested for their potential effect on P-gp expression. Three independent experiments were performed, in triplicate. Mouse IgG2a-PE was used as an isotype-matched negative control to estimate nonspecific binding of the PE-labelled anti-P-gp antibody (UIC2-PE).

#### 3.9.6. Evaluation of P-Glycoprotein Transport Activity

The effect of the tested compounds on P-gp activity was evaluated using rhodamine 123 (RHO 123) as a P-gp fluorescent substrate, and ZOS as a specific third-generation P-gp inhibitor. Indeed, several studies report the use of RHO 123 accumulation/efflux assays for the direct assessment of P-gp activity through the quantification of the intracellular fluorescence of the P-gp substrate [30]. Caco-2 cells were seeded in 24-well plates at a density of 60,000 cells/cm^2^ and, three days after seeding, and submitted to two different protocols of RHO 123 accumulation: 1) RHO 123 accumulation in cells pre-exposed to the compounds (20 µM) for 24 h; 2) RHO 123 accumulation in the presence of the tested compounds (20 µM) for 90 min, as previously described [50]. 

The first protocol, given the longer pre-incubation with the tested compounds for 24 h, aimed to assess whether potential compounds-induced increases (or decreases) in P-gp protein expression levels can be translated into potential increases (or decreases) in the pump activity. For that purpose, 3 days after seeding, Caco-2 cells were exposed to compounds (20 µM) in a fresh cell culture medium. Twenty-four hours after exposure, the cell culture medium containing the compounds was removed, the cells washed with HBSS (+/+) and incubated with RHO 123 in the absence of the tested compounds, and in the presence or absence of the specific P-gp inhibitor, zosuquidar (ZOS), accordingly to the following procedures:

RHO 123 accumulation under normal conditions (NA): pre-incubation with HBSS (+/+), for 30 min at 37 °C, followed by incubation with RHO 123 (5 μM) for 90 min, at 37 °C, in a humidified 5% CO_2_ and 95% O_2_ air atmosphere. 

RHO 123 accumulation in the presence of the P-gp inhibitor (IA): pre-incubation with ZOS (5 μM), prepared in HBSS (+/+), for 30 min at 37 °C, followed by incubation with RHO 123 (5 μM) for 90 min, at 37 °C, in a humidified 5% CO_2_ and 95% O_2_ air atmosphere. 

After the 90 min of incubation with RHO 123, the cells were washed twice with HBSS (-/-) and lysed with 1% Triton™ X100, for 30 min, in the dark, at room temperature. RHO 123 intracellular fluorescence was then measured at excitation/emission wavelengths of 485/528 nm, in a multi-well plate reader (PowerWave-X, BioTek Instruments, VT, USA) and expressed as fluorescence intensity (FI). The ratio between the FI after inhibited RHO 123 accumulation (IA) and the FI of non-inhibited RHO 123 accumulation (NA) was the parameter used for comparison, and the results were expressed as a percentage of control cells, as presented in the following equation.

(3) $$Rho123\;Accumulation=\frac{FI\;of\;RHO123\;accumulation\;under\;P-gp\;inhibition\;\left(IA\right)}{FI\;of\;RHO123\;accumulation\;under\;normal\;conditions\;\left(NA\right)}$$

Given the high autofluorescence of several of the tested compounds at the conditions used to measure RHO 123 fluorescence, only compounds **9**, **10**, **12**, **13** and **14** were able to be tested for their potential effect on P-gp activity. Five independent experiments were performed, in triplicate.

On the other hand, in the second protocol, the cells were exposed to the tested compounds 30 min prior to addition of the P-gp fluorescent substrate, RHO 123, and remained in contact with the cells only during the substrate accumulation period, thus aiming to detect immediate effects of the tested compounds on P-gp activity as a result of a direct P-gp activation or inhibition. For that purpose, three days after seeding, the cell culture medium was removed, and the cells submitted to the following procedures:

RHO 123 accumulation under normal conditions (NA): pre-exposure to compounds (20 μM), prepared in HBSS (+/+), for 30 min at 37 °C, followed by incubation with RHO 123 (5 μM) for 90 min, at 37 °C, in a humidified 5% CO_2_ and 95% O_2_ air atmosphere. Control cells were only exposed to RHO 123.

RHO 123 accumulation in the presence of the P-gp inhibitor (IA): simultaneous pre-exposure to compounds (20 μM) and ZOS (5 μM), both prepared in HBSS (+/+), for 30 min at 37 °C, followed by incubation with RHO 123 (5 μM) for 90 min, at 37 °C, in a humidified 5% CO_2_ and 95% O_2_ air atmosphere. Control cells were only exposed to ZOS and RHO 123. 

After the 90 min of incubation with RHO 123, the cells were washed twice with HBSS (-/-) and lysed with 1% Triton™ X100, for 30 min, in the dark, at room temperature. RHO 123 intracellular fluorescence was then measured at excitation/emission wavelengths of 485/528 nm, in a multi-well plate reader (PowerWave-X, BioTek Instruments, VT, USA) and expressed as fluorescence intensity (FI). The ratio between the FI after inhibited RHO 123 accumulation (IA) and the FI of non-inhibited RHO 123 accumulation (NA) was the parameter used for comparison, and the results were expressed as a percentage of control cells (Equation (3)). Given the high autofluorescence of several of the tested compounds at the conditions used to measure RHO 123 fluorescence, only compounds **9**, **10**, **12**, **13**, and **14** were able to be tested for their potential immediate effect on P-gp activity. Four independent experiments were performed, in triplicate.

#### 3.9.7. Statistical Analysis

The results of the cytotoxicity in the NIH/3T3 cell line were expressed as the mean ± standard deviation (SD), and the IC_50_ values were obtained by best fitting the dose-dependent inhibition curves in GraphPad Prism 5.03 for Windows software.

Statistical analysis of the P-glycoprotein assays described in 3.9.2. through 3.9.6. was performed using GraphPad Prism version 6.00 for Windows (GraphPad Software, San Diego, CA, USA). The normality of data distribution was evaluated with three different tests: KS normality test, D’Agostino and Pearson omnibus normality test and Shapiro–Wilk normality test. For data with parametric distribution, one-way ANOVA was used to perform the statistical comparisons, followed by the Dunnett’s multiple comparisons test. Details of the performed statistical analysis are described in each figure legend. In all cases, *p* values smaller than 0.05 were considered significant.

## 4. Conclusions

In this study, a set of thioxanthone derivatives showed their potential as antimicrobials and as antibiotic adjuvants. From the tested library, three compounds, **2**, **3** and **12**, display antibacterial activity against Gram-positive bacterial species, with **12** equally effective in the *Salmonella* strain. Furthermore, when tested for their potential as adjuvants of clinical important antibiotics towards resistant strains, compounds **10**, **11**, **12**, and **18** were able to decrease the MIC of cefotaxime in a clinical ESBL-producing *E. coli*, with **12** displaying a 16-fold reduction in the MIC of this third-generation cephalosporin, while not displaying antimicrobial activity. Moreover, compound **9** showed a decrease in the same magnitude in the VRE *E. faecalis* B3/101, and, as with compound **12**, it did not show antibacterial activity for this strain. This may suggest an underlying capacity to neutralize their specific resistance mechanism, although further studies are needed to clarify this subject.

The compounds were also tested for their potential to inhibit the efflux of EB, which may translate into the inhibition of bacterial efflux pumps. In this assay, five compounds, **2**, **3**, **7**, **11**, and **13**, showed an increase in fluorescence compared to the positive controls, and were shown not to be fluorescent themselves. These results could imply that these thioxanthones can act as bacterial efflux pump inhibitors. However, there are other mechanisms behind the inhibition of EB efflux, such as membrane destabilization. The lack of MIC for the concentrations tested may be a suggestion that these compounds do not interfere with membrane stability, but studies on bacterial membrane depolarization, or gene and protein expression, are warranted to clarify this issue.

Regarding the biofilm and QS assays, tolerance and virulence mechanisms related to efflux pump inhibition, thioxanthones have also displayed promising results. It is noteworthy that compound **3**, which displayed a high biofilm inhibition in both tested strains and QS inhibition in EZF+CV026, was previously highlighted in the EB efflux inhibition in both tested strains. Adding to these activities, this compound was proven to be non-cytotoxic in the tested cell line, emphasizing its potential to be employed as an antimicrobial adjuvant, or to be further studied as an antimicrobial itself, as previous studies have shown its activity against resistant strains [12].

The pyrimidine derivative **8** was also successful in inhibiting EB efflux in SE03 and QS, while not displaying cytotoxicity. Its capability of decreasing the MIC of cefotaxime in a resistant *E. coli* strain also makes it an interesting compound, due to difficulties in reaching Gram-negative strains.

The sulfamide derivatives **12** and **13** also show potential with different profiles. Compound **12** was effective as a Gram-positive antimicrobial and as a Gram-negative antimicrobial adjuvant; it was effective in inhibiting biofilm formation in the resistant *S. aureus* strain, but it also displayed cytotoxicity, warranting further modifications to improve its safety profile. Additionally, this compound displayed inhibition of P-gp (ABCB1). Compound **13**, on the other hand, was effective at inhibiting EB efflux in the *Salmonella* strain tested, and in inhibiting biofilm formation of the resistant strain, while not displaying cytotoxicity. As with compound **12**, it also showed inhibitory activity concerning P-gp. In contrast, the 1-bromo precursor **14** was found to be an activator of P-glycoprotein, with a slight but immediate activity.

Previous studies with compounds **2–7** described **2** is a competitive inhibitor of P-gp and **3**–**7** as non-competitive inhibitors [11]. P-gp belongs to the ATP-binding cassette family, a family which is also present in bacteria, but does not acquire the relevance of the MFS or RND families. However, the fact that compounds **2**, **3**, **7**, and **13** present action in both the inhibition of P-gp and EB efflux, can either mean that these compounds can act as dual human and bacterial efflux pump inhibitors, or that they are inhibitors of ABC pumps, which warrants further studies regarding the bacterial efflux pump inhibition.

Overall, these results show the interesting profile of thioxanthone derivatives, opening the possibility of their use as antimicrobials or in the circumvention of resistance mechanisms. So far, to the best of our knowledge, thioxanthones have not yet been described for their potential in these activities, to the best of our knowledge. Future studies should focus on the combination of these compounds, for potential synergy studies. The clarification on the precise mechanisms through which they exert their action or to which, if any, efflux pumps they bind to is an opportunity for further research. Docking results performed suggest their favorable affinity towards the most relevant targets, AcrAB-TolC and NorA, but deeper insights will allow to conclude with more certainty about the specific action of these compounds.

## Figures and Tables

**Table 1:** Synthetic procedures and structures of the compounds. Reagents and conditions (a) X = Cl (**1**), amine, K_2_CO_3_, CuI, MeOH, 100 °C, 48 h, sealed flask; (b) X = Br (**14**), amine, K_2_CO_3_, CuI, MeOH, 100 °C, 48 h, sealed flask; (c) KBr, H_3_PO_4_, CuI, nitrobenzene, 48 h, reflux.

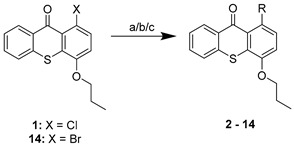		
**Compound**	**R**	**Procedure**
**2**	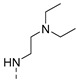	a
**3**	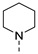	a
**4**	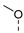	a
**5**	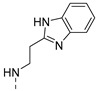	a
**6**	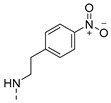	a
**7**	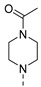	a
**8**	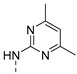	a
**9**	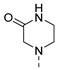	a
**10**	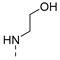	a
**11**	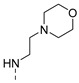	a
**12**	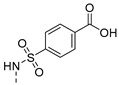	a, b
**13**	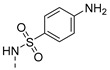	b
**14**	Br	c
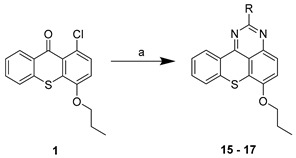		
**Compound**	**R**	**Procedure**
**15**	H	a
**16**	NH_2_	a
**17**	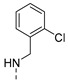	a
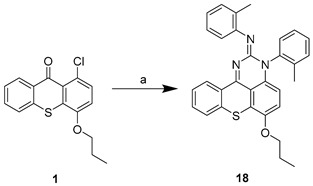		

**Table 2:** Docking results for the thioxanthones **2**–**18** and reference compounds against bacterial and mammalian efflux pumps.

Compound	Docking Scores								
	AcrB		AcrA		TolC	NorA		P-gp	
	SBS	HT	HH	LD		BCR	CS	TMD	NBD
**1**	−6.3	17.5	−5.4	−4.2	−6.2	−5.2	−4.9	−5.7	−6.6
**2**	−6.1	−1.0	−5.4	−5.4	−6.3	−5.6	−4.9	−7.2	−5.2
**3**	−6.9	−5.0	−5.6	−4.7	−6.8	−3.9	−5.7	−8.5	−5.3
**4**	−6.5	−5.1	−5.4	−5.2	−6.2	−5.3	−5.3	−7.1	−5.7
**5**	−7.5	4.2	−6.2	−6.3	−7.5	−7.8	−6.2	−8.6	−5.2
**6**	−7.4	−3.4	−6.5	−6.6	−6.9	−7.4	−5.3	−9.2	−6.3
**7**	−7.6	4.8	−5.7	−4.9	−7.7	−2.3	−5.5	−8.0	−5.6
**8**	−8.1	−2.5	−6.0	−5.2	−7.3	−6.2	−5.0	−9.2	−6.3
**9**	−7.3	−3.6	−5.8	−4.8	−6.9	−4.0	−5.8	−8.2	−5.6
**10**	−7.2	−5.7	−5.8	−5.3	−6.7	−6.1	−5.7	−7.2	−5.7
**11**	−7.5	1.6	−6.1	−5.7	−7.2	−6.1	−5.7	−7.4	−5.9
**12**	−8.2	−5.7	−6.9	−6.4	−7.8	−5.5	−5.7	−8.9	−6.6
**13**	−7.8	−4.8	−6.5	−6.0	−7.1	−6.2	−5.1	−9.2	−5.8
**14**	−6.3	−4.7	−5.4	−5.3	−6.4	−4.7	−5.7	−7.4	−5.5
**15**	−6.5	−5.6	−5.7	−5.7	−6.6	−5.8	−5.4	−7.3	−5.1
**16**	−7.4	−5.7	−5.8	−6.1	−7.0	−5.5	−5.6	−8.0	−6.7
**17**	−7.9	−6.2	−6.9	−5.6	−9.0	−5.2	−6.5	−9.2	−7.2
**18**	−8.5	32.9	−6.8	−5.7	−8.4	4.0	−7.4	−10.4	−6.0
**D13-9001**	−9.7	26.5	−6.2	−5.1	−7.4	-	-	-	-
**Doxorubicin**	−8.9	15.4	−7.2	−5.6	−7.2	-	-	−8.3	−7.5
**MBX-3132**	−7.9	2.9	−7.9	−6.2	−7.7	-	-	-	-
**Minocycline**	−8.7	26.7	−6.2	−5.4	−7.7	-	-	−6.8	−6.3
**PA** **βN**	−7.1	−4.7	−5.8	−4.9	−7.1	−9.4	−5.3	-	-
**Reserpine**	−8.7	10.9	5.6	4.6	−7.5	1.0	−4.6	-	-
***trans*(*E*)-flupentixol**	-	-	-	-	-	−3.2	−4.6	-	-
**Verapamil**	-	-	-	-	-	-	-	−7.1	−4.1

**SBS**: Substrate-binding site; **HT**: Hydrophobic trap; **HH**: Helical hairpin; **LD**: Lipoyl domain; **BCR**: Binding core region; **CS**: Cytoplasmic side; **TMD**: Transmembrane domain; **NBD**: Nucleotide binding domain.

**Table 3:** Minimum inhibitory concentrations of the compounds in the antibacterial activity assay and synergy with antibiotics.

Compound	Antibacterial Activity						Synergy with Antimicrobials	
	Minimum Inhibitory Concentration (MIC) (µM)							
	*E. coli* ATCC 25922	*P. aeruginosa* ATCC 27853	*E. faecalis* ATCC 29212	*S. aureus* ATCC 29213	*S. aureus* 272123	SE03	*E. coli* SA/2	*E. faecalis* B3/101
							CTX MIC = 1124 (512 µg/mL)	VAN MIC = 707 (1024 µg/mL)
							CTX + Compound ^1^	VAN + Compound ^2^
**2**	-	-	83 (32 µg/mL)	83 (32 µg/mL)	>100	>100	-	-
**3**	-	-	>100	>100	>100	>100	-	-
**4**	-	-	-	-	>100	>100	-	-
**5**	-	-	-	-	>100	>100	-	-
**6**	-	-	-	-	>100	>100	-	-
**7**	-	-	-	-	>100	>100	-	-
**8**	>100	>100	>100	>100	>100	>100	281 (128 µg/mL)	707 (1024 µg/mL)
**9**	>100	>100	>100	>100	>100	>100	1124 (512 µg/mL)	44 (64 µg/mL)
**10**	>100	>100	>100	>100	>100	>100	281 (128 µg/mL)	707 (1024 µg/mL)
**11**	>100	>100	>100	>100	>100	>100	1124 (512 µg/mL)	707 (1024 µg/mL)
**12**	>100	>100	34 (16 µg/mL)	34 (16 µg/mL)	>100	50	70 (32 µg/mL)	707 (1024 µg/mL)
**13**	>100	>100	>100	>100	>100	>100	1124 (512 µg/mL)	707 (1024 µg/mL)
**14**	>100	>100	>100	>100	>100	>100	1124 (512 µg/mL)	707 (1024 µg/mL)
**15**	>100	>100	>100	>100	>100	>100	1124 (512 µg/mL)	707 (1024 µg/mL)
**16**	>100	>100	>100	>100	>100	>100	1124 (512 µg/mL)	707 (1024 µg/mL)
**17**	>100	>100	>100	>100	>100	>100	1124 (512 µg/mL)	707 (1024 µg/mL)
**18**	>100	>100	>100	>100	>100	>100	281 (128 µg/mL)	707 (1024 µg/mL)

^1^ All compounds were kept at the highest concentration tested in the antibacterial activity (64 µg/mL), considering that none showed a direct inhibitory effect on *E. coli*. ^2^ As compound **12** revealed antibacterial activity against *E. faecalis*, the synergy with vancomycin was determined in the presence of the highest concentration that did not affect bacterial growth when the compound was used alone (8 µg/mL). All the other compounds were kept at the highest concentration tested in the antibacterial activity assay (64 µg/mL). Compounds **2**–**7** were previously tested for their antimicrobial activity and synergy in these strains [12]. **CTX**: cefotaxime; **VAN**: vancomycin. **SE03**: *Salmonella enterica* serovar Typhimurium SL1344 (SE03).

**Table 4:** Relative fluorescence index (RFI) of tested derivatives.

Compound	RFI ± SD	
	*S. aureus* 272123	SE03
**2**	0.75 ± 0.08	−0.02 ± 0.04
**3**	1.50 ± 0.04	0.67 ± 0.01
**4**	0.11 ± 0.26	−0.10 ± 0.05
**5**	−0.13 ± 0.04	−0.20 ± 0.01
**6**	0.09 ± 0.11	−0.20 ± 0.01
**7**	−0.10 ± 0.02	0.49 ± 0.11
**8**	0.24 ± 0.07	0.74 ± 0.08
**9**	0.14 ± 0.02	1.54 ± 0.09
**10**	4.71 ± 0.78	6.00 ± 0.80
**11**	0.89 ± 0.06	1.37 ± 0.054
**12**	0.29 ± 0.19	−0.21 ± 0.07
**13**	−0.18 ± 0.05	1.23 ± 0.18
**14**	1.13 ± 0.13	0.87 ± 0.11
**15**	−0.07 ± 0.50	0.54 ± 0.11
**16**	0.96 ± 0.27	7.52 ± 0.01
**17**	9.69 ± 0.95	25.51 ± 2.93
**18**	0.20 ± 0.03	0.20 ± 0.02
**Reserpine**	0.50 ± 0.04	---
**CCCP**	---	0.23 ± 0.04

**SD**: Standard deviation; **SE03**: *Salmonella enterica* serovar Typhimurium SL1344; **CCCP**: carbonyl cyanide 3-chlorophenylhydrazone.

**Table 5:** Biofilm and quorum sensing inhibition of the thioxanthones tested.

Compound	Biofilm Inhibition (%) ± SD		Quorum Sensing Inhibition (mm) ± SD		
	*S. aureus* ATCC 29213	*S. aureus* 272123	*S. marcescens*	wt85	EZF + CV026
**2**	0.53 ± 1.05 ^1^	81.11 ± 1.18 ^3^	0	0	0
**3**	93.35 ± 0.86 ^2^	93.49 ± 0.33 ^4^	0	0	31 ± 0.8
**4**	0.17 ± 0.83 ^2^	47.92 ± 1.08 ^4^	0	0	0
**5**	0.37 ± 0.47 ^2^	0 ^4^	0	0	0
**6**	0 ^2^	0 ^4^	0	0	0
**7**	0 ^2^	47.07 ± 7.54 ^4^	0	0	30 ± 0.5
**8**	0 ^2^	0 ^5^	0	0	32 ± 0.1
**9**	5.18 ± 1.02 ^2^	31.48 ± 8.99 ^5^	0	0	0
**10**	0 ^2^	55.17 ± 4.20 ^5^	0	0	0
**11**	0 ^2^	20.17 ± 4.00 ^4^	31 ± 0.8	0	0
**12**	53.32 ± 7.81 ^2^	96.50 ± 0.67 ^5^	0	0	0
**13**	0.42 ± 1.36 ^2^	72.04 ± 6.66 ^4^	0	0	51 ± 0.1
**14**	0 ^2^	2.46 ± 0.72 ^4^	0	0	0
**15**	0 ^2^	75.95 ± 2.06 ^4^	32 ± 0.5	0	50 ± 0.1
**16**	0 ^2^	73.59 ± 1.80 ^4^	0	0	29 ± 0.8
**17**	0 ^2^	6.32 ± 4.32 ^4^	0	0	49 ± 0.5
**18**	19.68 ±7.46 ^2^	1.62 ± 2.43 ^5^	0	0	0
**Reserpine**	^1^ 30.37 ± 7.32 ^2^ 22.29 ± 5.10	^3^ 90.34 ± 3.35 ^4^ 72.10 ± 1.54 ^5^ 93.76 ± 2.01	ND	ND	ND
**PMZ**	ND	ND	18 ± 0.8	40 ± 0.1	41 ± 0.5

^1–5^ The value of the positive control in each different assay; **wt85**: *C. violaceum* wild-type 85; **EZF**: *Sphingomonas paucimobilis* Ezf 10–17; **CV026**: *C. violaceum* CV026; SD: Standard deviation. PMZ: Promethazine. ND: not determined.

## Data Availability

The data presented in this study are available on request from the corresponding author.

## Supplementary Materials

The following are available online at https://www.mdpi.com/article/10.3390/ph14060572/s1. Figure S1. Main ^1^H and ^13^C signals for the 1-nitrogen substituted thioxanthones 8–14; Figure S2. Main connectivities found in the HMBC for the thioxanthone scaffold used in this work; Figure S3. Evaluation of the fluorescence of compound **2** alone and in combination with EB; Figure S4. Evaluation of the fluorescence of compound **3** alone and in combination with EB; Figure S5. Evaluation of the fluorescence of compound **9** alone and in combination with EB; Figure S6. Evaluation of the fluorescence of compound **10** alone and in combination with EB; Figure S7. Evaluation of the fluorescence of compound **11** alone and in combination with EB; Figure S8. Evaluation of the fluorescence of compound **14** alone and in combination with EB; Figure S9. Evaluation of the fluorescence of compound **15** alone and in combination with EB; Figure S10. Evaluation of the fluorescence of compound **16** alone and in combination with EB; Figure S11. Evaluation of the fluorescence of compound **17** alone and in combination with EB; Figure S12. Molecular visualization in the SBS of AcrB. (A) Interaction between compound **3** and the SBS; (B) and (C) Different perspectives of the interactions between compound **7** and the SBS; (D) and (E) Different perspectives of the interactions between compound **8** and the SBS; (G) and (H) Different perspectives of the interactions between **13** and the SBS; Figure S13. ^1^H NMR and ^13^C NMR for compound **8**; Figure S14. ^1^H NMR and ^13^C NMR for compound **9**; Figure S15. Electrospray ESI data for compound **9**; Figure S16. ^1^H NMR and ^13^C NMR for compound **10**; Figure S17. Electrospray ESI data for compound **10**; Figure S18. ^1^H NMR and ^13^C NMR for compound **11**; Figure S19. Electrospray ESI data for compound **11**; Figure S20. ^1^H NMR and ^13^C NMR for compound **12**; Figure S21. Electrospray ESI data for compound **12**; Figure S22. ^1^H NMR and ^13^C NMR for compound **13**; Figure S23. Electrospray ESI data for compound **13**; Figure S24. ^1^H NMR and ^13^C NMR for compound **14**; Figure S25. GC-MS spectra of compound **14**.

## References

[1] Robey R.W., Pluchino K.M., Hall M.D., Fojo A.T., Bates S.E., Gottesman M.M. (2018). Revisiting the role of ABC transporters in multidrug-resistant cancer. Nat. Rev. Cancer.

[2] Palmeira A., Sousa E., Vasconcelos M.H., Pinto M. (2012). Three Decades of P-gp Inhibitors: Skimming Through Several Generations and Scaffolds. Curr. Med. Chem..

[3] Nanayakkara A.K., Follit C.A., Chen G., Williams N.S., Vogel P.D., Wise J.G. (2018). Targeted inhibitors of P-glycoprotein increase chemotherapeutic-induced mortality of multidrug resistant tumor cells. Sci. Rep..

[4] Durães F., Pinto M., Sousa E. (2018). Medicinal Chemistry Updates on Bacterial Efflux Pump Modulators. Curr. Med. Chem..

[5] Nikaido H., Takatsuka Y. (2009). Mechanisms of RND multidrug efflux pumps. Biochim. Biophys. Acta.

[6] Kumar S., Mukherjee M.M., Varela M.F. (2013). Modulation of Bacterial Multidrug Resistance Efflux Pumps of the Major Facilitator Superfamily. Int. J. Bacteriol..

[7] Alcalde-Rico M., Hernando-Amado S., Blanco P., Martínez J.L. (2016). Multidrug Efflux Pumps at the Crossroad between Antibiotic Resistance and Bacterial Virulence. Front. Microbiol..

[8] Alav I., Sutton J.M., Rahman K.M. (2018). Role of bacterial efflux pumps in biofilm formation. J. Antimicrob. Chemother..

[9] Soto S.M. (2013). Role of efflux pumps in the antibiotic resistance of bacteria embedded in a biofilm. Virulence.

[10] Pinto M.M.M., Palmeira A., Fernandes C., Resende D.I.S.P., Sousa E., Cidade H., Tiritan M.E., Correia-da-Silva M., Cravo S. (2021). From Natural Products to New Synthetic Small Molecules: A Journey through the World of Xanthones. Molecules.

[11] Palmeira A., Vasconcelos M.H., Paiva A., Fernandes M.X., Pinto M., Sousa E. (2012). Dual inhibitors of P-glycoprotein and tumor cell growth: (Re)discovering thioxanthones. Biochem. Pharmacol..

[12] Bessa L.J., Palmeira A., Gomes A.S., Vasconcelos V., Sousa E., Pinto M., Martins da Costa P. (2015). Synergistic Effects Between Thioxanthones and Oxacillin Against Methicillin-Resistant *Staphylococcus aureus*. Microb. Drug Resist..

[13] Durães F., Resende D.I.S.P., Palmeira A., Szemerédi N., Pinto M.M.M., Spengler G., Sousa E. (2021). Xanthones Active against Multidrug Resistance and Virulence Mechanisms of Bacteria. Antibiotics.

[14] Grossman O., Azerraf C., Gelman D. (2006). Palladium Complexes Bearing Novel Strongly Bent Trans-Spanning Diphosphine Ligands:  Synthesis, Characterization, and Catalytic Activity. Organometallics.

[15] Durães F., Silva P.M.A., Novais P., Amorim I., Gales L., Esteves C.I.C., Guieu S., Bousbaa H., Pinto M., Sousa E. (2021). Tetracyclic Thioxanthene Derivatives: Studies on Fluorescence and Antitumor Activity. Molecules.

[16] Gales L., Damas A.M. (2005). Xanthones-A Structural Perspective. Curr. Med. Chem..

[17] Freitas V.L.S., Gomes J.R.B., Gales L., Damas A.M., Ribeiro da Silva M.D.M.C. (2010). Experimental and Computational Studies on the Structural and Thermodynamic Properties of Two Sulfur Heterocyclic Keto Compounds. J. Chem. Eng. Data.

[18] Kaatz G.W., Moudgal V.V., Seo S.M., Kristiansen J.E. (2003). Phenothiazines and thioxanthenes inhibit multidrug efflux pump activity in *Staphylococcus aureus*. Antimicrob. Agents Chemother..

[19] Shi X., Chen M., Yu Z., Bell J.M., Wang H., Forrester I., Villarreal H., Jakana J., Du D., Luisi B.F. (2019). In situ structure and assembly of the multidrug efflux pump AcrAB-TolC. Nat. Commun..

[20] Yan N. (2013). Structural advances for the major facilitator superfamily (MFS) transporters. Trends Biochem. Sci..

[21] Aron Z., Opperman T.J. (2018). The hydrophobic trap—the Achilles heel of RND efflux pumps. Res. Microbiol..

[22] Zárate S.G., Morales P., Świderek K., Bolanos-Garcia V.M., Bastida A. (2019). A Molecular Modeling Approach to Identify Novel Inhibitors of the Major Facilitator Superfamily of Efflux Pump Transporters. Antibiotics.

[23] Bessa L.J., Barbosa-Vasconcelos A., Mendes A., Vaz-Pires P., Martins da Costa P. (2014). High prevalence of multidrug-resistant Escherichia coli and Enterococcus spp. in river water, upstream and downstream of a wastewater treatment plant. J. Water Health.

[24] Anand N., Hitchings G.H. (1983). Sulfonamides: Structure-Activity Relationships and Mechanism of Action. Inhibition of Folate Metabolism in Chemotherapy: The Origins and Uses of Co-trimoxazole.

[25] Mouwakeh A., Kincses A., Nové M., Mosolygó T., Mohácsi-Farkas C., Kiskó G., Spengler G. (2019). *Nigella sativa* essential oil and its bioactive compounds as resistance modifiers against *Staphylococcus aureus*. Phytother. Res..

[26] Sharma A., Gupta V.K., Pathania R. (2019). Efflux pump inhibitors for bacterial pathogens: From bench to bedside. Indian J. Med. Res..

[27] Parai D., Banerjee M., Dey P., Mukherjee S.K. (2020). Reserpine attenuates biofilm formation and virulence of *Staphylococcus aureus*. Microb. Pathog..

[28] Zimmermann S., Klinger-Strobel M., Bohnert J.A., Wendler S., Rödel J., Pletz M.W., Löffler B., Tuchscherr L. (2019). Clinically Approved Drugs Inhibit the *s* Multidrug NorA Efflux Pump and Reduce Biofilm Formation. Front. Microbiol..

[29] Gajdács M., Spengler G. (2019). The Role of Drug Repurposing in the Development of Novel Antimicrobial Drugs: Non-Antibiotic Pharmacological Agents as Quorum Sensing-Inhibitors. Antibiotics.

[30] Silva R., Palmeira A., Carmo H., Barbosa D., Gameiro M., Gomes A., Paiva M., Sousa E., Pinto M., Bastos M. (2014). P-glycoprotein induction in Caco-2 cells by newly synthetized thioxanthones prevents paraquat cytotoxicity. Arch. Toxicol..

[31] Takara K., Hayashi R., Kokufu M., Yamamoto K., Kitada N., Ohnishi N., Yokoyama T. (2009). Effects of nonsteroidal anti-inflammatory drugs on the expression and function of P-glycoprotein/MDR1 in Caco-2 cells. Drug Chem. Toxicol..

[32] Sheldrick G.M. (2008). A short history of SHELX. Acta Crystallogr. A.

[33] Eicher T., Cha H.-j., Seeger M.A., Brandstätter L., El-Delik J., Bohnert J.A., Kern W.V., Verrey F., Grütter M.G., Diederichs K. (2012). Transport of drugs by the multidrug transporter AcrB involves an access and a deep binding pocket that are separated by a switch-loop. Proc. Natl. Acad. Sci. USA.

[34] Mikolosko J., Bobyk K., Zgurskaya H.I., Ghosh P. (2006). Conformational Flexibility in the Multidrug Efflux System Protein AcrA. Structure.

[35] Koronakis V., Sharff A., Koronakis E., Luisi B., Hughes C. (2000). Crystal structure of the bacterial membrane protein TolC central to multidrug efflux and protein export. Nature.

[36] Sussman J.L., Lin D., Jiang J., Manning N.O., Prilusky J., Ritter O., Abola E.E. (1998). Protein Data Bank (PDB): Database of three-dimensional structural information of biological macromolecules. Acta Crystallogr. D Biol. Crystallogr..

[37] Trott O., Olson A.J. (2010). AutoDock Vina: Improving the speed and accuracy of docking with a new scoring function, efficient optimization, and multithreading. J. Comput. Chem..

[38] Waterhouse A., Bertoni M., Bienert S., Studer G., Tauriello G., Gumienny R., Heer F.T., de Beer T.A.P., Rempfer C., Bordoli L. (2018). SWISS-MODEL: Homology modelling of protein structures and complexes. Nucleic Acids Res..

[39] (2017). UniProt: The universal protein knowledgebase. Nucleic Acids Res..

[40] Kim Y., Chen J. (2018). Molecular structure of human P-glycoprotein in the ATP-bound, outward-facing conformation. Science.

[41] Ward A.B., Szewczyk P., Grimard V., Lee C.W., Martinez L., Doshi R., Caya A., Villaluz M., Pardon E., Cregger C. (2013). Structures of P-glycoprotein reveal its conformational flexibility and an epitope on the nucleotide-binding domain. Proc. Natl. Acad. Sci. USA.

[42] Li J., Jaimes K.F., Aller S.G. (2014). Refined structures of mouse P-glycoprotein. Protein Sci..

[43] Szewczyk P., Tao H., McGrath A.P., Villaluz M., Rees S.D., Lee S.C., Doshi R., Urbatsch I.L., Zhang Q., Chang G. (2015). Snapshots of ligand entry, malleable binding and induced helical movement in P-glycoprotein. Acta Crystallogr. D Biol. Crystallogr..

[44] Seeliger D., de Groot B.L. (2010). Ligand docking and binding site analysis with PyMOL and Autodock/Vina. J. Comput. Aided Mol. Des..

[45] Gajdács M., Spengler G. (2020). Standard operating procedure (SOP) for disk diffusion-based quorum sensing inhibition assays. Acta Pharm. Hung..

[46] CLSI (2018). Methods for Dilution Antimicrobial Susceptibility Tests for Bacteria that Grow Aerobically.

[47] Ferreira R.J., Kincses A., Gajdács M., Spengler G., dos Santos D.J.V.A., Molnár J., Ferreira M.-J.U. (2018). Terpenoids from *Euphorbia pedroi* as Multidrug-Resistance Reversers. J. Nat. Prod..

[48] Repetto G., del Peso A., Zurita J.L. (2008). Neutral red uptake assay for the estimation of cell viability/cytotoxicity. Nat. Protoc..

[49] Skehan P., Storeng R., Scudiero D., Monks A., McMahon J., Vistica D., Warren J.T., Bokesch H., Kenney S., Boyd M.R. (1990). New colorimetric cytotoxicity assay for anticancer-drug screening. J. Natl. Cancer Inst..

[50] Silva V., Gil-Martins E., Rocha-Pereira C., Lemos A., Palmeira A., Puthongking P., Sousa E., de Lourdes Bastos M., Remião F., Silva R. (2020). Oxygenated xanthones as P-glycoprotein modulators at the intestinal barrier: In vitro and docking studies. Med. Chem. Res..

