# Supplementary material for: Antimicrobial Activity of a Library of Thioxanthones and Their Potential as Efflux Pump Inhibitors
Source: Pharmaceuticals (Basel). 2021 Jun 15;14(6):572. doi: 10.3390/ph14060572 (PMC8232621; doi:10.3390/ph14060572)
Supplement: Supplementary file 1 [file pharmaceuticals-14-00572-s001.zip › pharmaceuticals-1256940-supplementary.pdf]

Supporting Information

Antimicrobial activity of a library of thioxanthones and their potential as efflux pump inhibitors

Fernando Durães [1,2], Andreia Palmeira [1,2], Bárbara Cruz [3], Joana Freitas-Silva [2,4], Nikoletta Szemerédi [5], Luís Gales [6,7], Paulo Martins da Costa [2,4], Fernando Remião [3], Renata Silva [3], Madalena Pinto [1,2], Gabriella Spengler [5] *, Emília Sousa [1,2] *

[1] Laboratory of Organic and Pharmaceutical Chemistry, Department of Chemical Sciences, Faculty of Pharmacy, University of Porto, Rua de Jorge Viterbo Ferreira, 228, 4050-313 Porto, Portugal; fduraes5@gmail.com (F. D.); apalmeira@ff.up.pt (A.P.).

[2] CIIMAR-Interdisciplinary Centre of Marine and Environmental Research, University of Porto, Novo Edifício do Terminal de Cruzeiros do Porto de Leixões, Avenida General Norton de Matos, S/N, 4450-208 Matosinhos, Portugal; madalena@ff.up.pt (M.P.), joanafreitasdasilva@gmail.com (J.F.-S.).

[3] UCIBIO-REQUIMTE, Laboratory of Toxicology, Faculty of Pharmacy, University of Porto, Rua de Jorge Viterbo Ferreira 228, 4050-313 Porto, Portugal; up201607633@med.up.pt (B.C.); remiao@ff.up.pt (F.R.), rsilva@ff.up.pt (R.S.)

[4] ICBAS – Institute of Biomedical Sciences Abel Salazar, Universidade do Porto, Rua de Jorge Viterbo Ferreira 228, 4050-313 Porto, Portugal; pmcosta@icbas.up.pt (P.M.C.).

[5] Department of Medical Microbiology and Immunobiology, Faculty of Medicine, University of Szeged, Dóm tér 10, 6720 Szeged, Hungary; szemeredi.nikoletta@med.u-szeged.hu (N. S.).

[6] Department of Molecular Biology, ICBAS - Instituto de Ciências Biomédicas Abel Salazar, University of Porto, Porto, Portugal; lgales@ibmc.up.pt (L.G.)

[7] Bioengineering & Synthetic Microbiology, I3S – Instituto de Investigação e Inovação em Saúde, University of Porto, Porto, Portugal

* Correspondence: spengler.gabriella@med.u-szeged.hu (G. S.); esousa@ff.up.pt (E. S.).

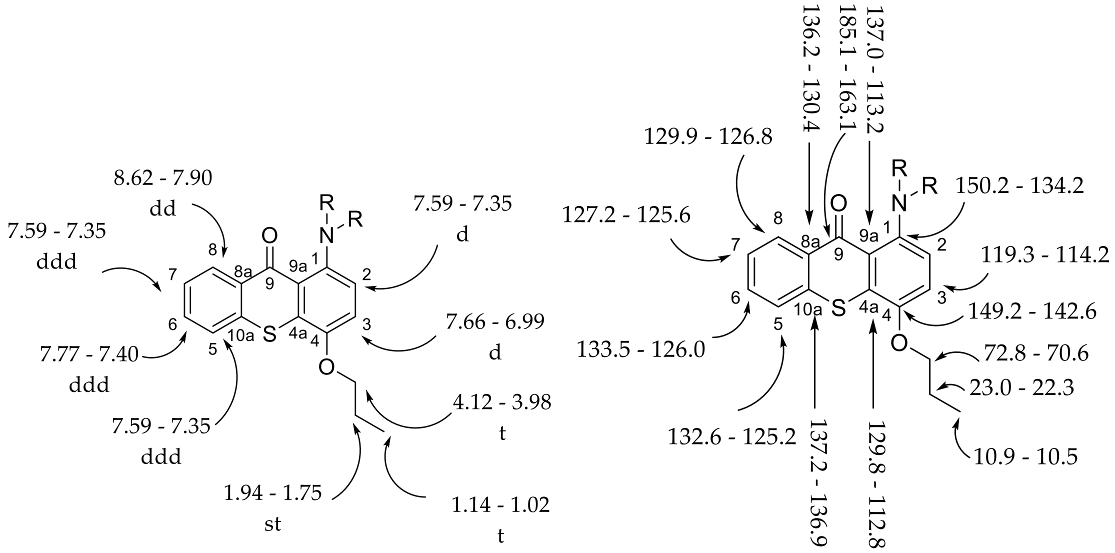

Figure S1. Main ¹H (left) and ¹³C (right) signals for the 1-nitrogen substituted thioxanthones **8-14**.

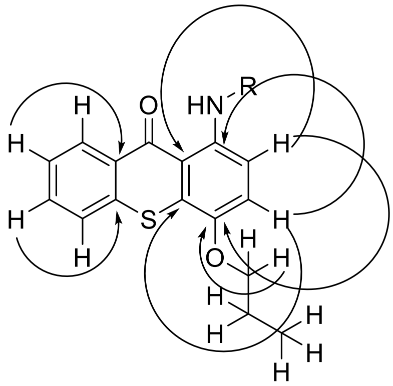

Figure S2. Main connectivities found in the HMBC for the thioxanthone scaffold used in this work.

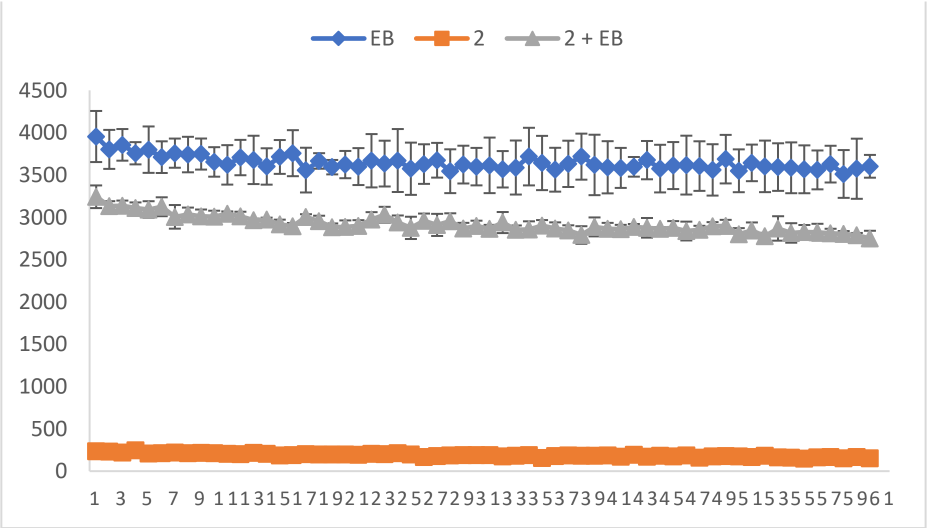

Figure S3. Evaluation of the fluorescence of compound **2** alone and in combination with EB.

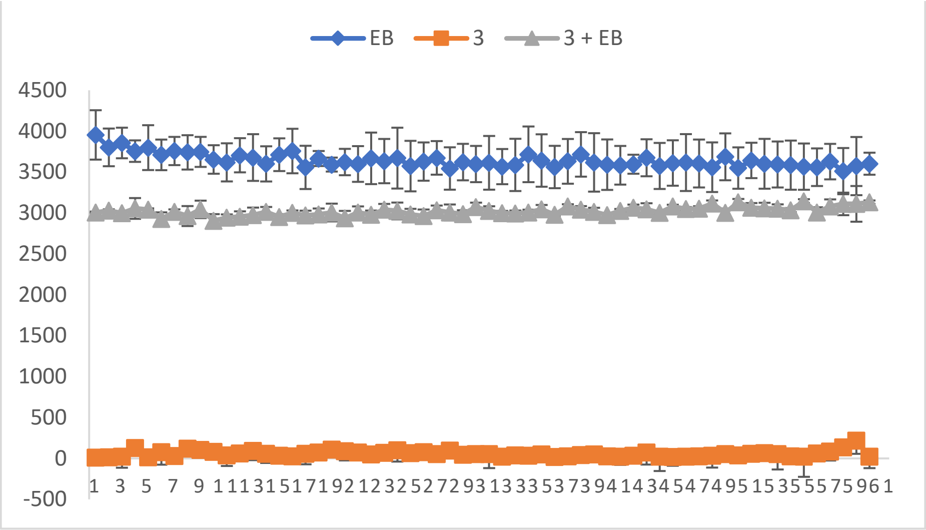

Figure S4. Evaluation of the fluorescence of compound **3** alone and in combination with EB.

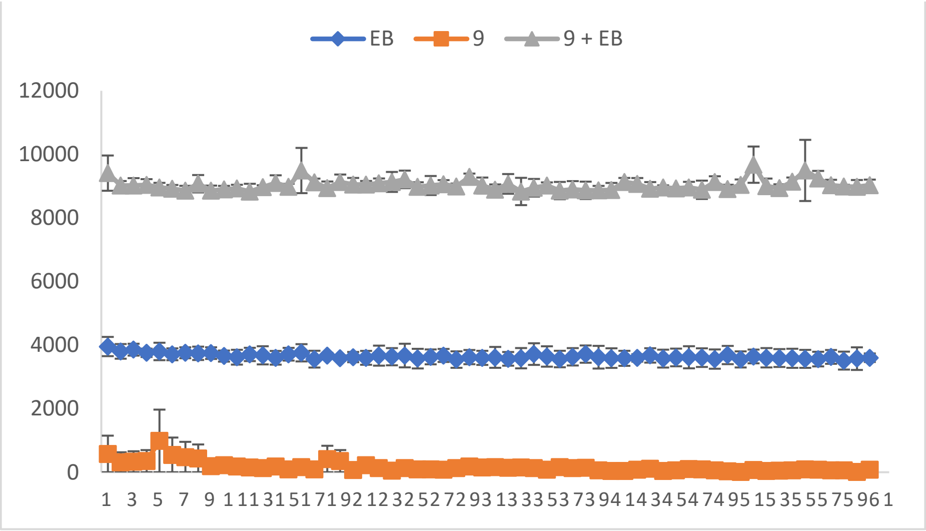

Figure S5. Evaluation of the fluorescence of compound **9** alone and in combination with EB.

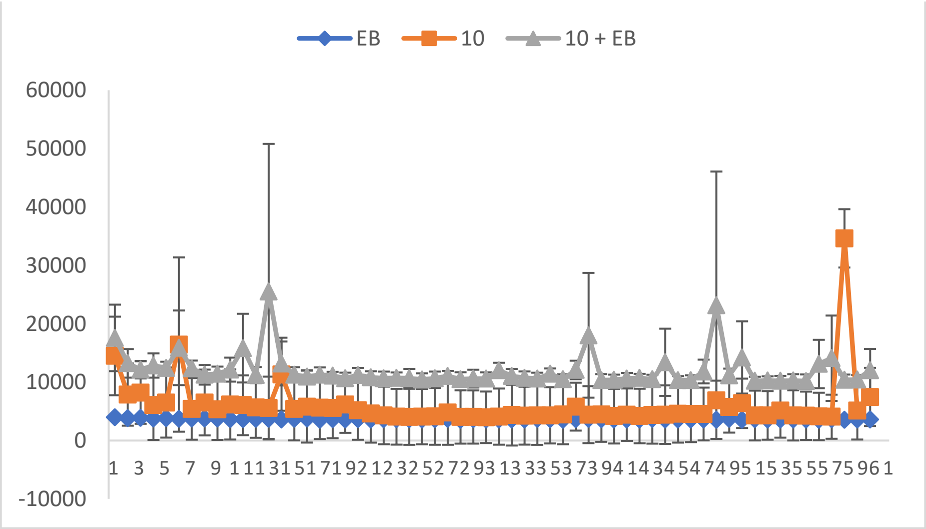

Figure S6. Evaluation of the fluorescence of compound **10** alone and in combination with EB.

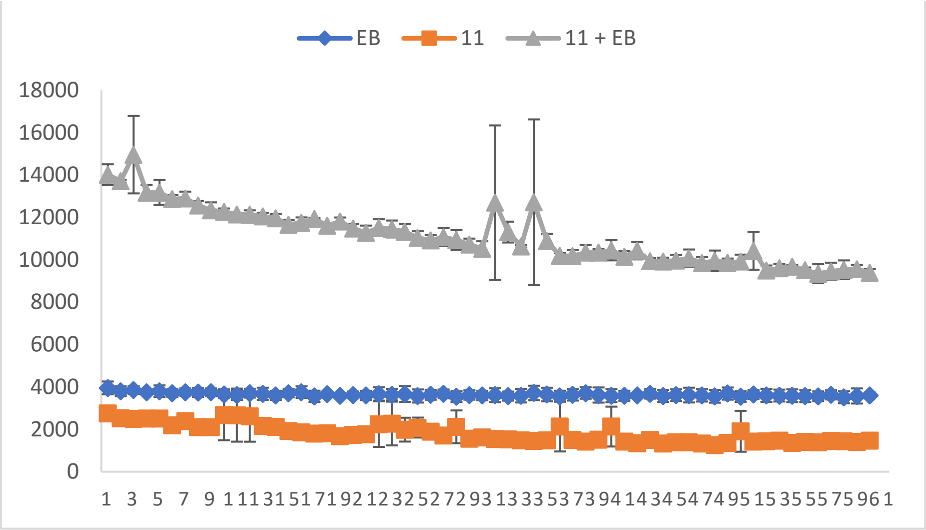

Figure S7. Evaluation of the fluorescence of compound **11** alone and in combination with EB.

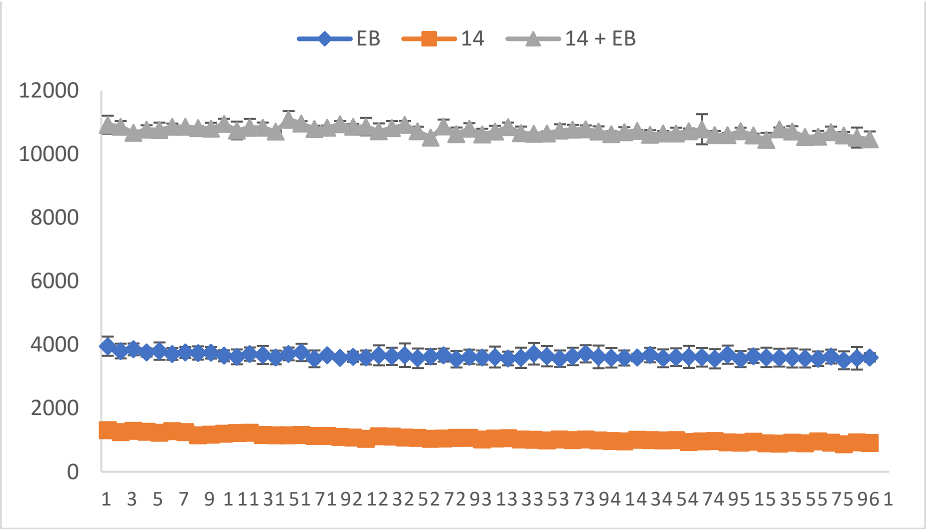

Figure S8. Evaluation of the fluorescence of compound **14** alone and in combination with EB.

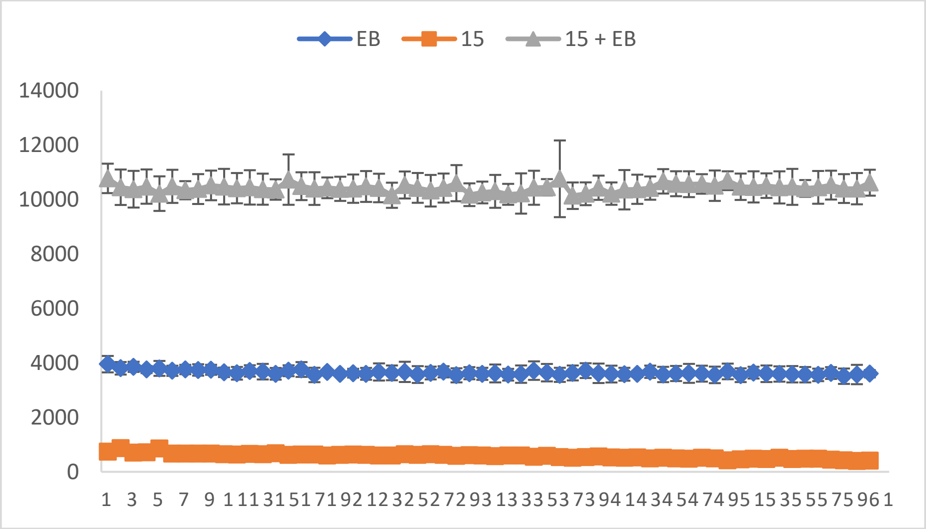

Figure S9. Evaluation of the fluorescence of compound **15** alone and in combination with EB.

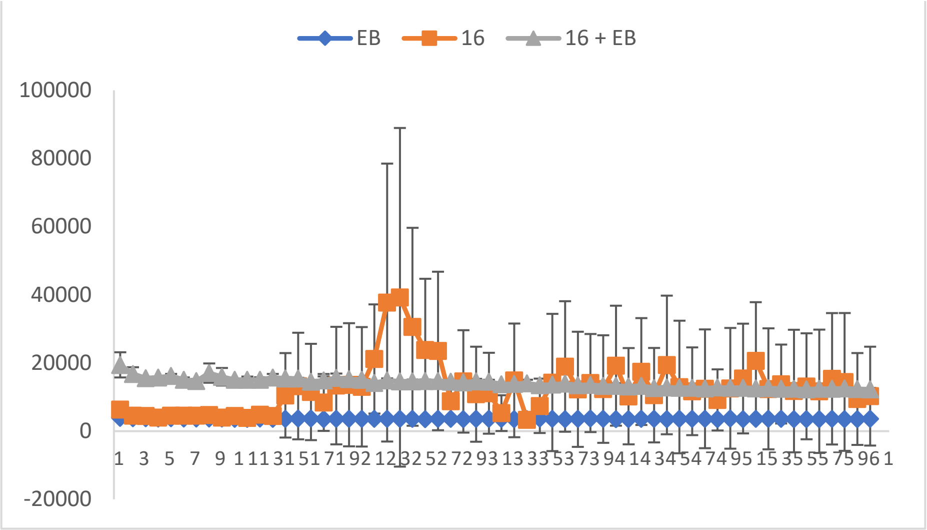

Figure S10. Evaluation of the fluorescence of compound **16** alone and in combination with EB.

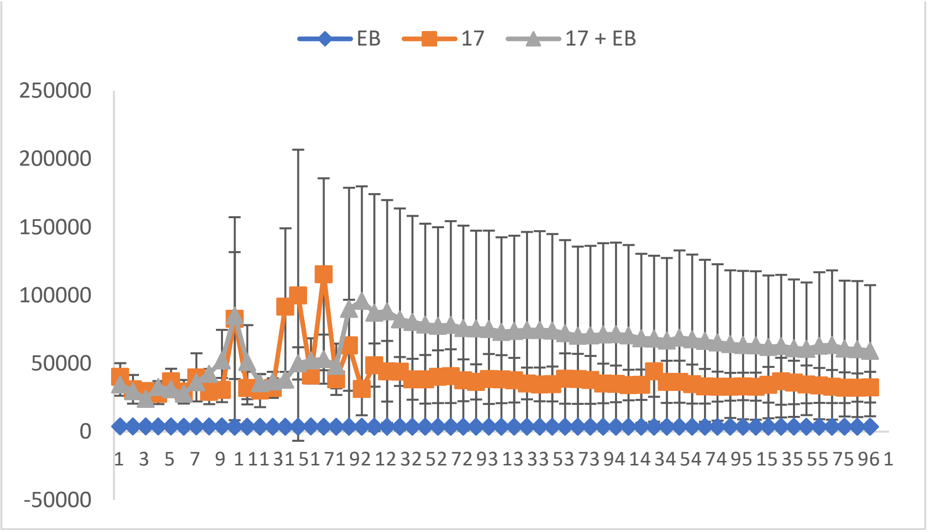

Figure S11. Evaluation of the fluorescence of compound **17** alone and in combination with EB.

Molecular visualization of compounds 3, 7, 8, and 13 into the substrate-binding site of AcrB:

Compounds **3**, **7**, **8** and **13** were visualized in the SBS of AcrB. As it can be seen in **Figure 4**, **D**, the compounds are predicted to bind in the approximate site, but it is suggested that they interact with different residues. This is clarified when the compounds are analyzed individually.

Compound **3** (**Figure S11**, **A**) is predicted to only interact with Arg-620, through a hydrogen bond between the oxygen in the propoxy chain and a nitrogen in this residue. While it is true that all the compounds present the propoxy chain, it is also noteworthy that compound **3** has the simplest and most flexible substituent out of all this group of compounds, which can possibly allow it to get to this site within the SBS.

Compound **7** (**Figure S12**, **B** and **C**), hydrogen bonds can be seen between the carbonyl in C-9 and Gln-176, the oxygen in the propoxy chain in C-4 and Asn-274, and between the ketone of the substituent in C-1 and Gln-89.

For compound **8** (**Figure S12**, **D** and **E**), it can be noted that the nitrogens within the pyrimidine moiety form dipole interactions with Gln-89 and Gln-176, and the carbonyl in C-9 of the thioxanthone moiety forms a hydrogen bond with the amine of Gln-89. Additionally, there is a T-shaped π-π interaction between the pyrimidine substituent and Phe-615.

Lastly, for compound **13** (**Figure S12**, **F** and **G**), many different hydrogen interactions can be seen. The oxygen in the propoxy chain interacts with Gln-89, like **7** and **8.** The oxygens in the sulfamide interact with Thr-87 and Arg-620, and the amine directly bonded to the aromatic ring

in the substituent can also interact with Ser-46. The carbonyl in C-9 can also interact with a water molecule, that can show hydrogen interactions with Gln-176 and Leu-177.

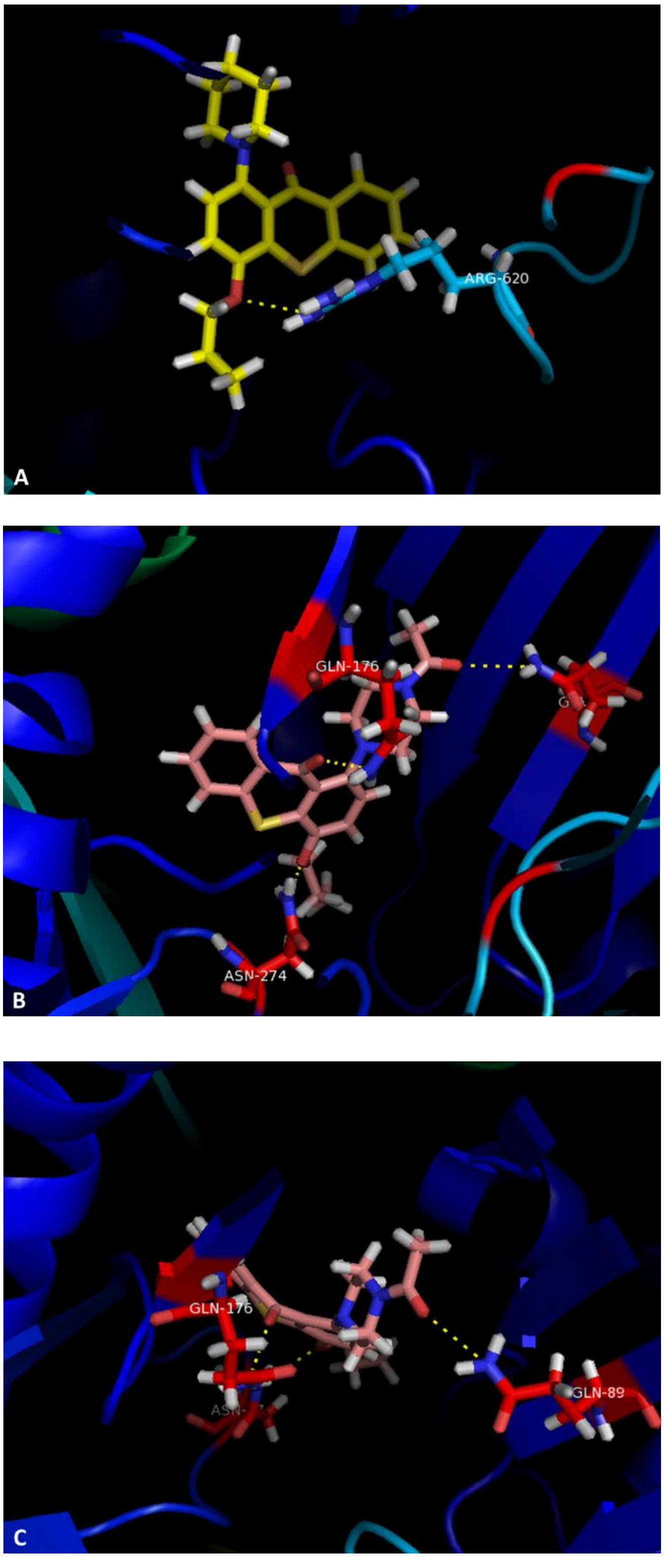

Figure S12. Molecular visualization in the SBS of AcrB. (A) Interaction between compound **3** and the SBS; (B) and (C) Different perspectives of the interactions between compound **7** and the SBS; (D) and (E) Different perspectives of the interactions between compound **8** and the SBS; (G) and (H) Different perspectives of the interactions between **13** and the SBS.

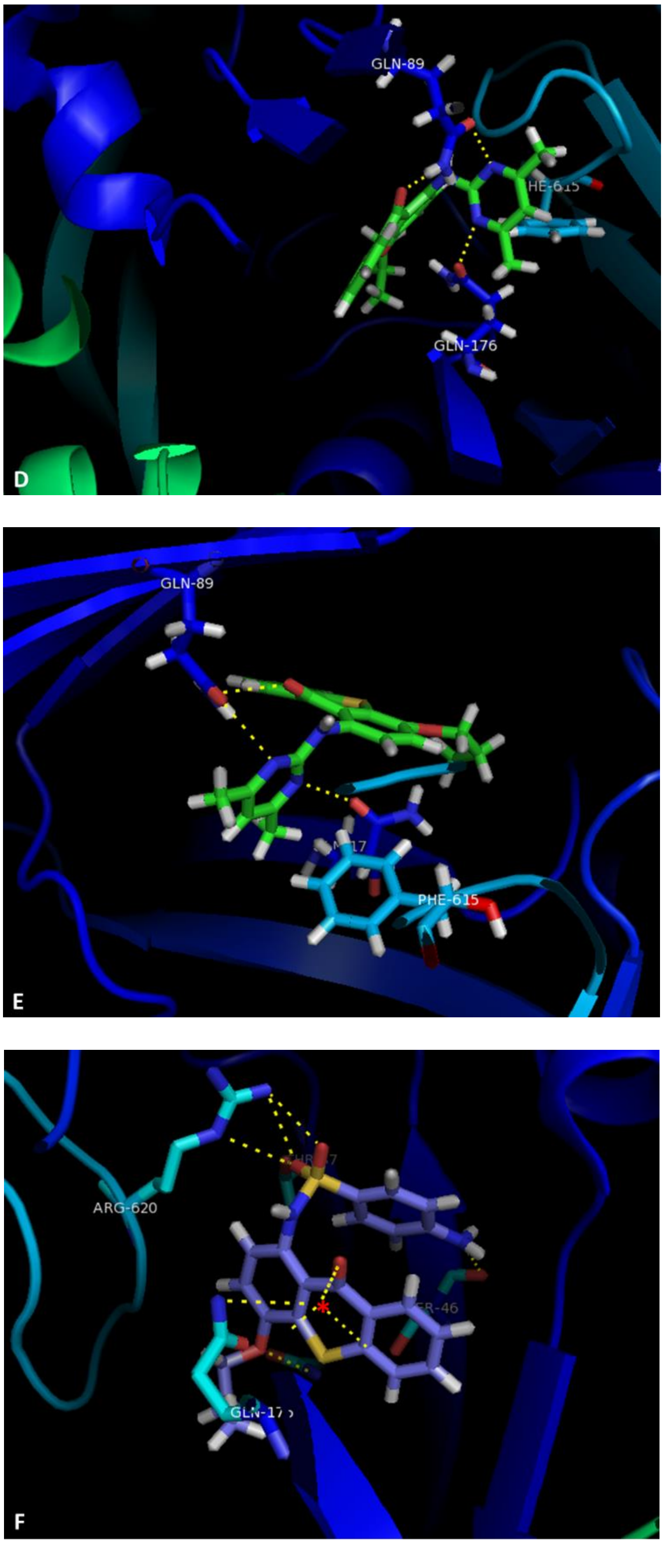

Figure S12. (cont'd)

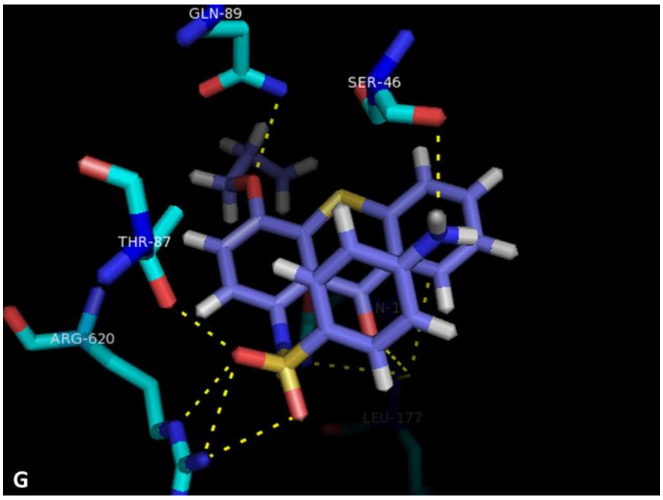

Figure S12. (cont'd)

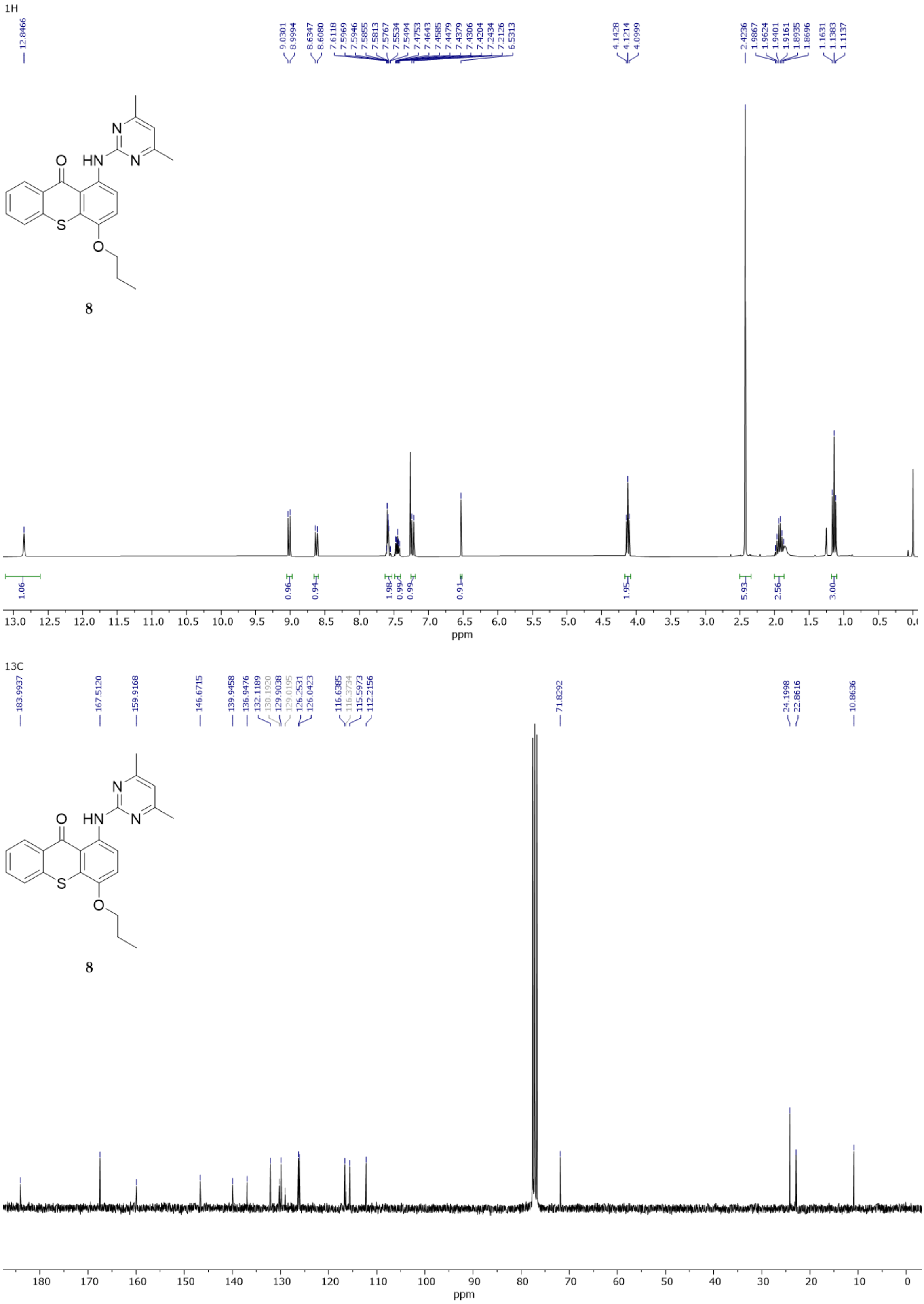

Figure S13. ^{1}H NMR (top, 300.13 MHz, $CDCl_3$) and ^{13}C NMR (bottom, 75.48 MHz, $CDCl_3$) for compound **8**.

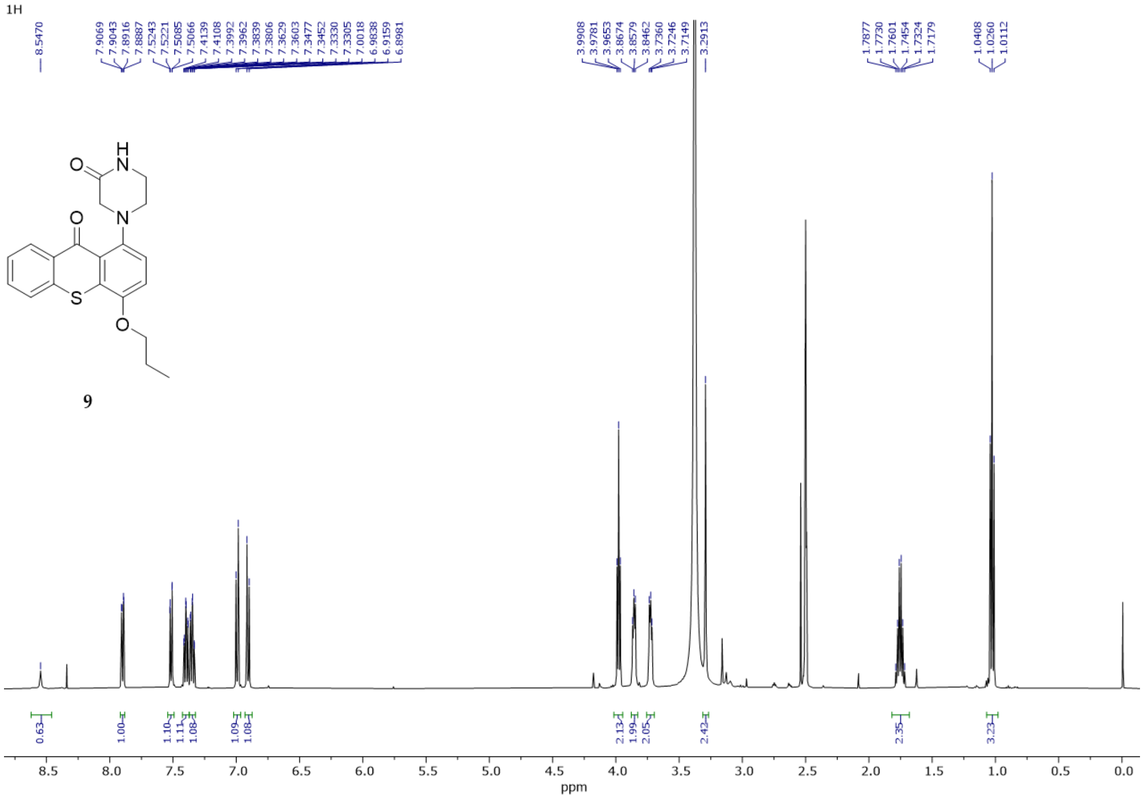

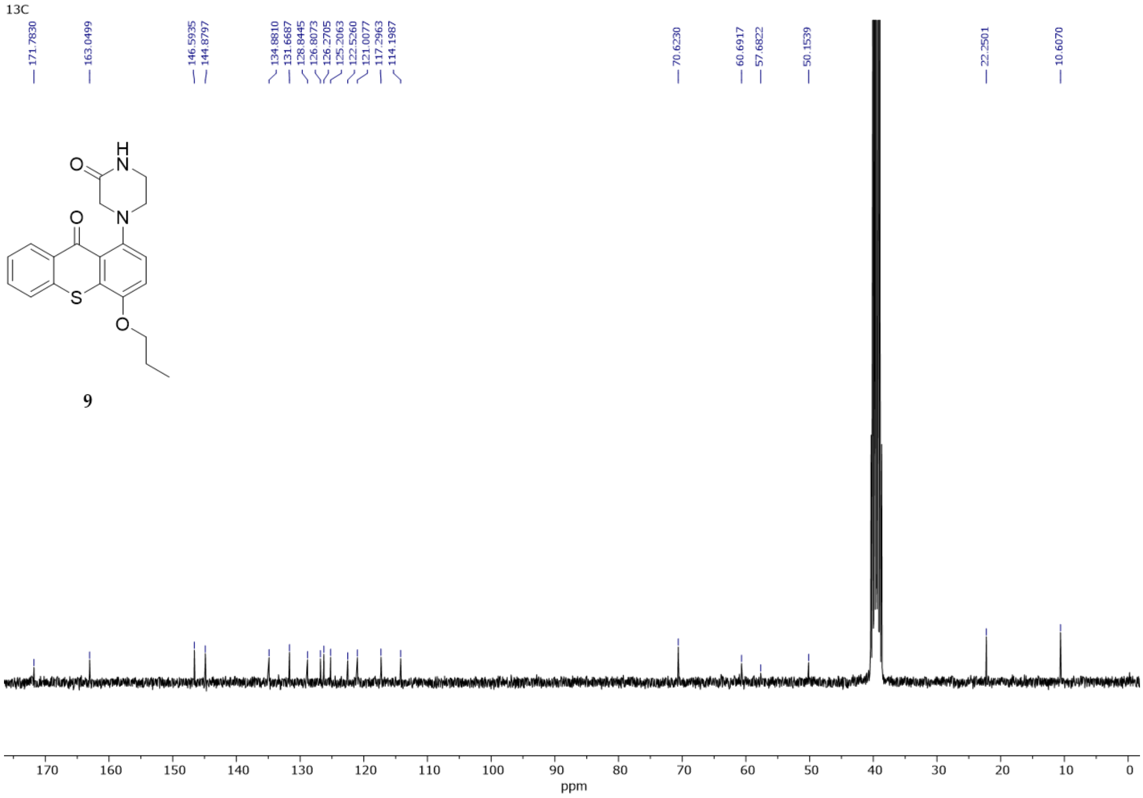

Figure S14. ^{1}H NMR (top, 500.13 MHz, DMSO) and ^{13}C NMR (bottom, 75.48 MHz, DMSO) for compound **9**.

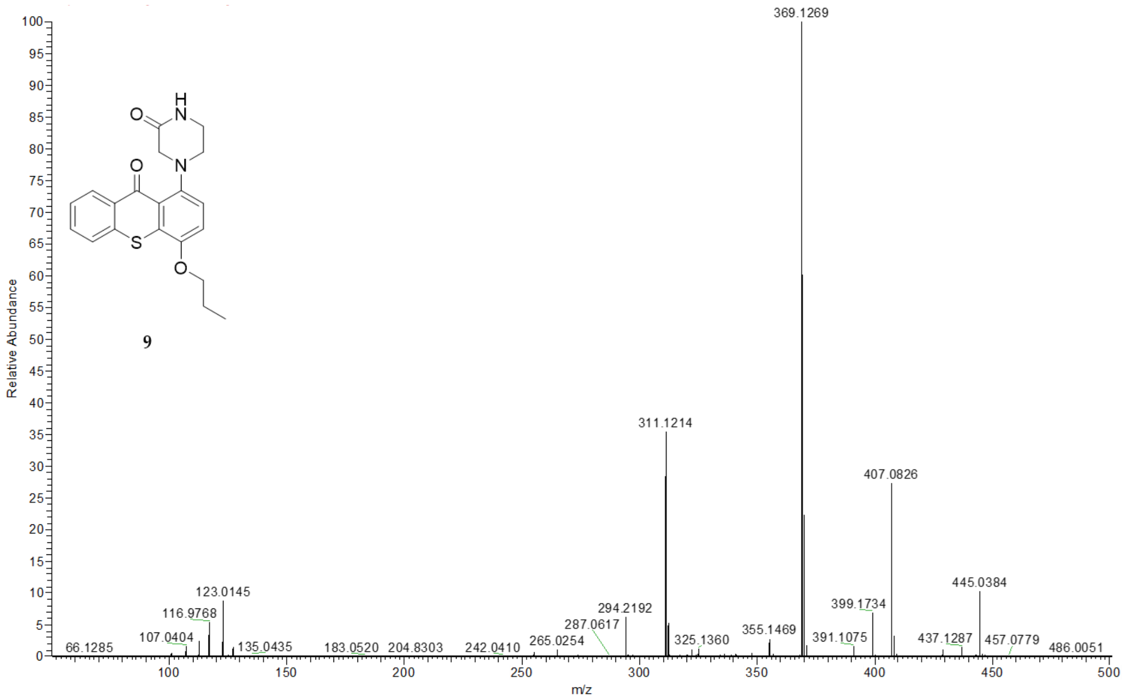

Meas. m/z	Formula	m/z	err [ppm]
369.1269	$C_{20}H_{21}N_2O_3S$	369.1273	-1.08

Figure S15. Electrospray ESI data for compound **9**.

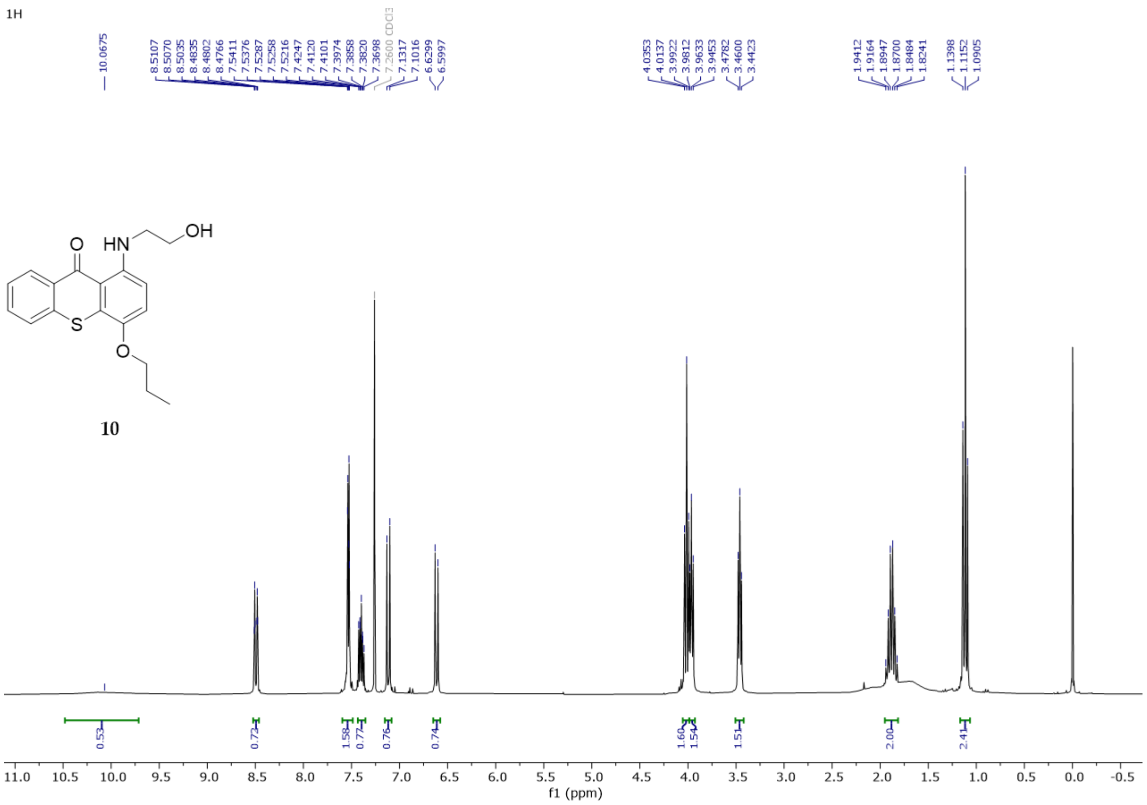

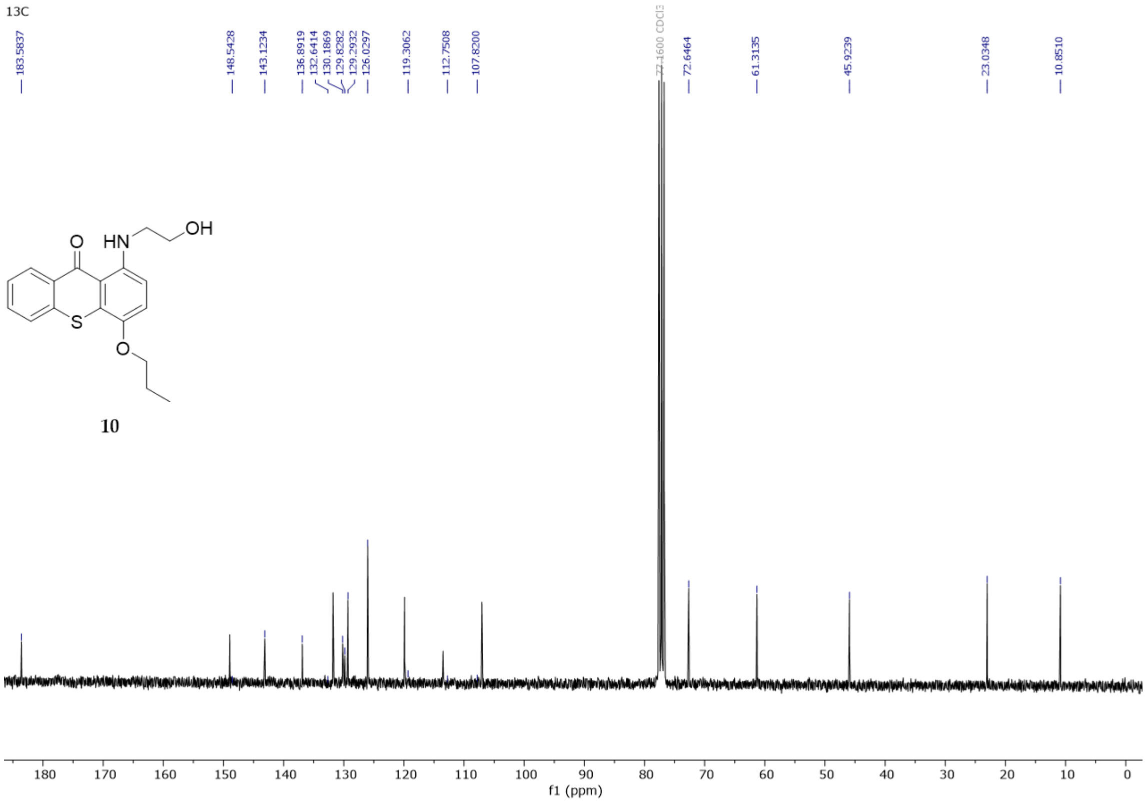

Figure S16. ^{1}H NMR (top, 300.13 MHz, $CDCl_3$) and ^{13}C NMR (bottom, 75.48 MHz, $CDCl_3$) for compound **10**.

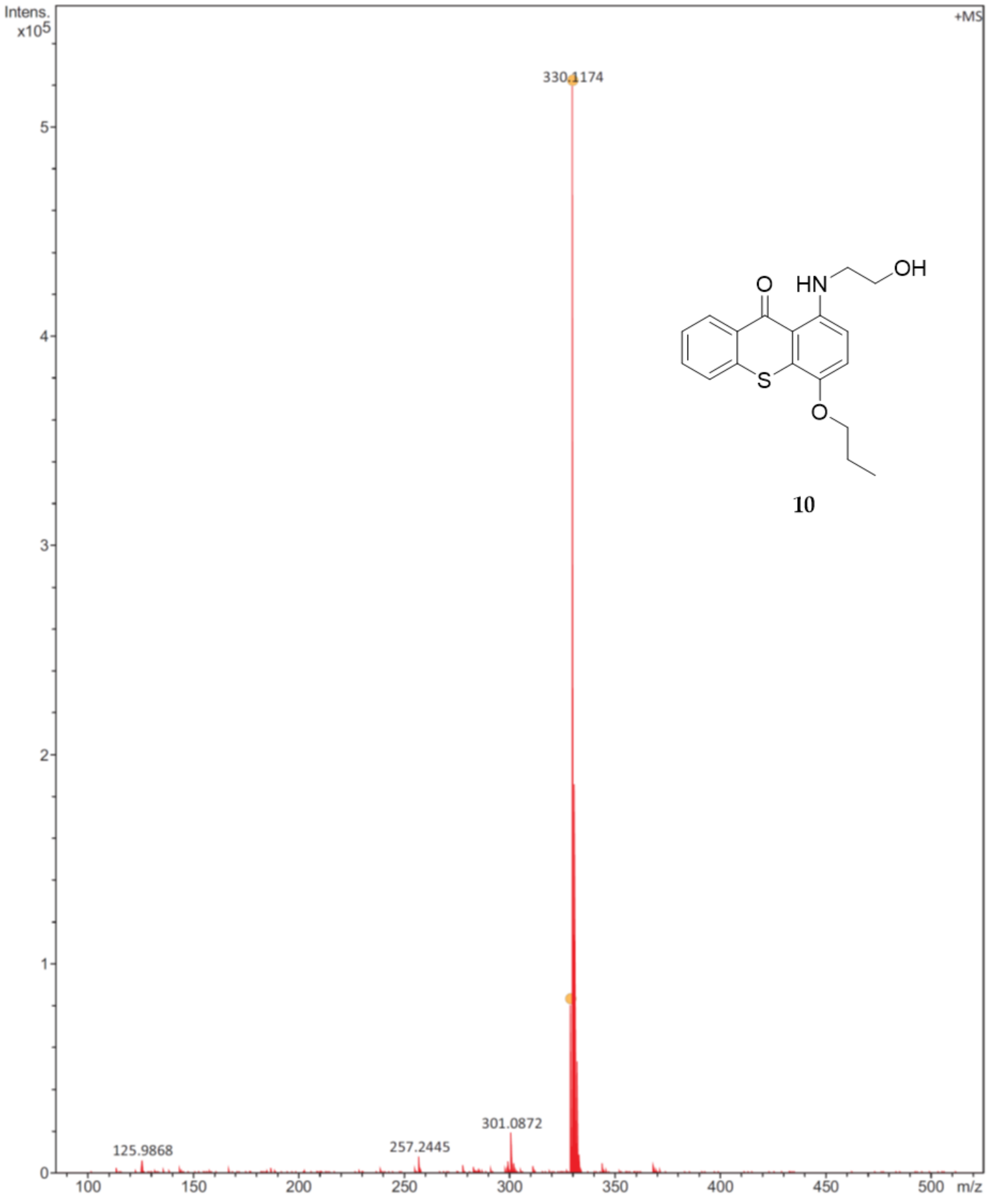

Meas. m/z	Formula	m/z	err [ppm]
329.1066	$C_{18}H_{19}NO_3S$	329.10866	4.40

Figure S17. Electrospray ESI data for compound **10**.

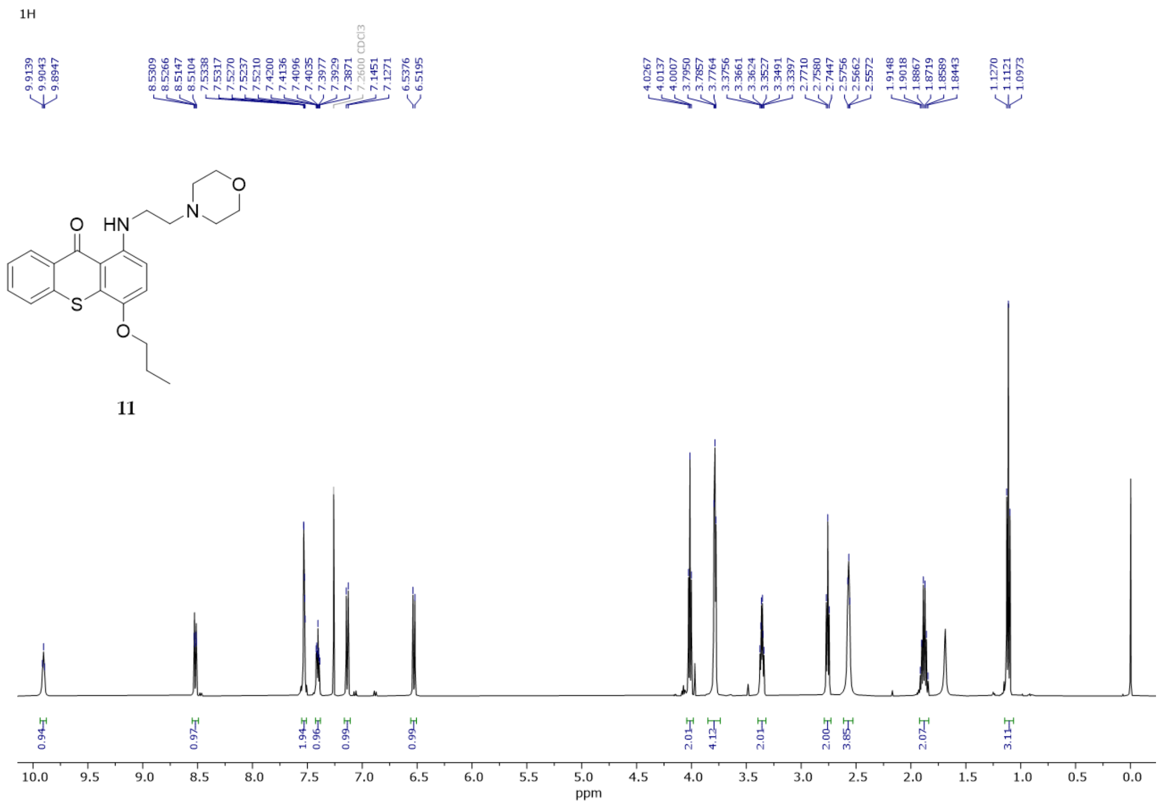

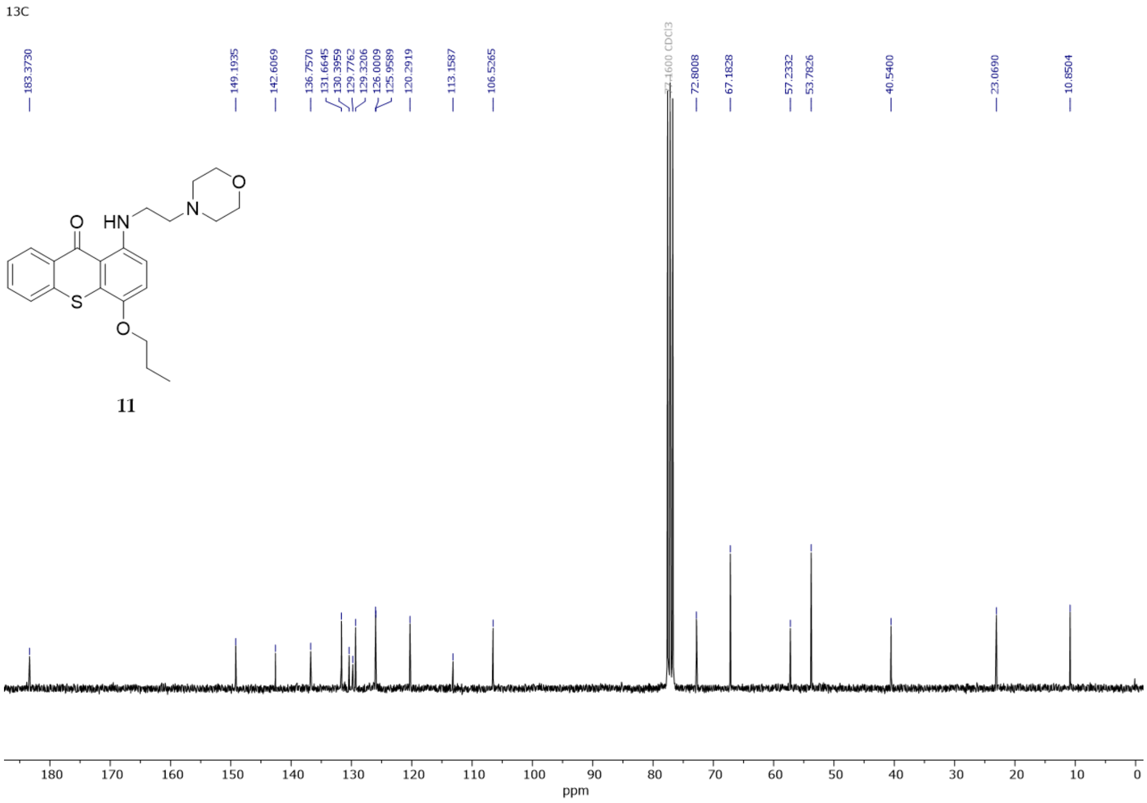

Figure S18. ^{1}H NMR (top, 500.13 MHz, $CDCl_3$) and ^{13}C NMR (bottom, 75.48 MHz, $CDCl_3$) for compound **11**.

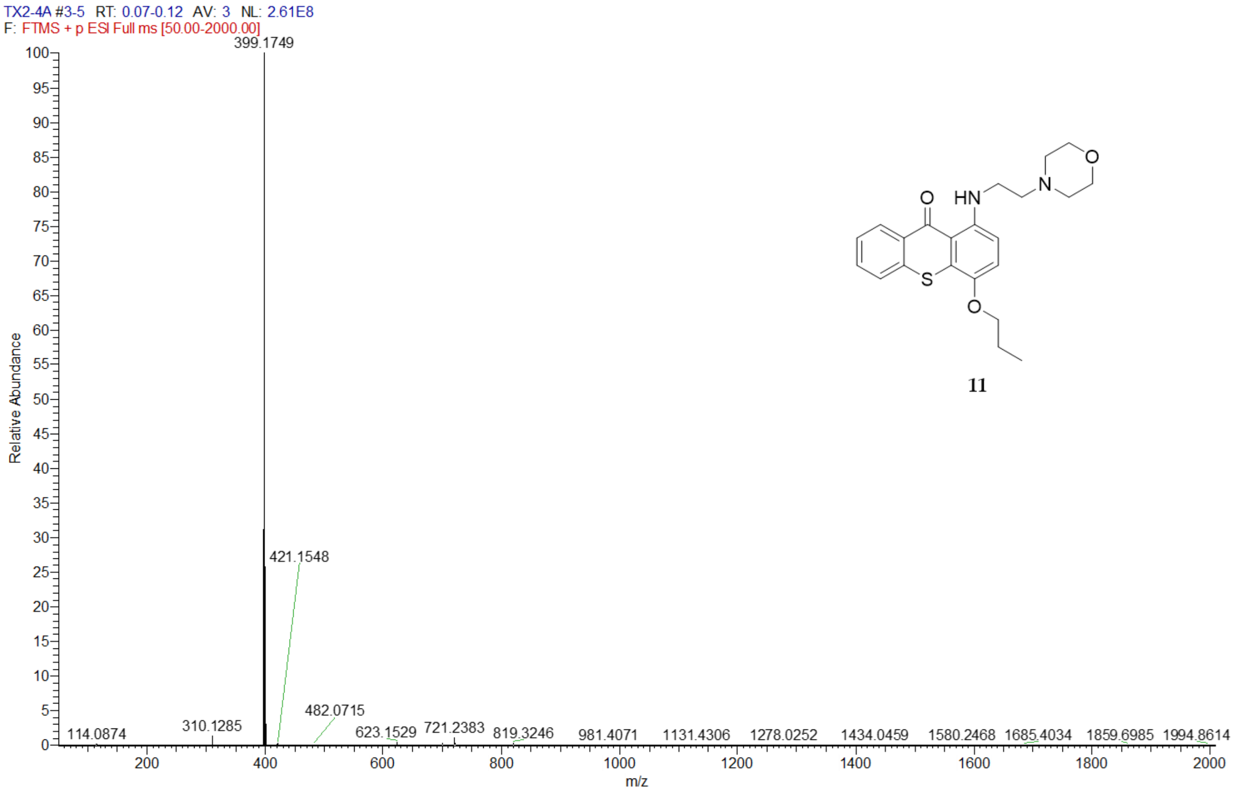

Meas. m/z	Formula	m/z	err [ppm]
399.1749	$C_{22}H_{27}N_2O_3S$	399.1742	1.75

Figure S19. Electrospray ESI data for compound **11**.

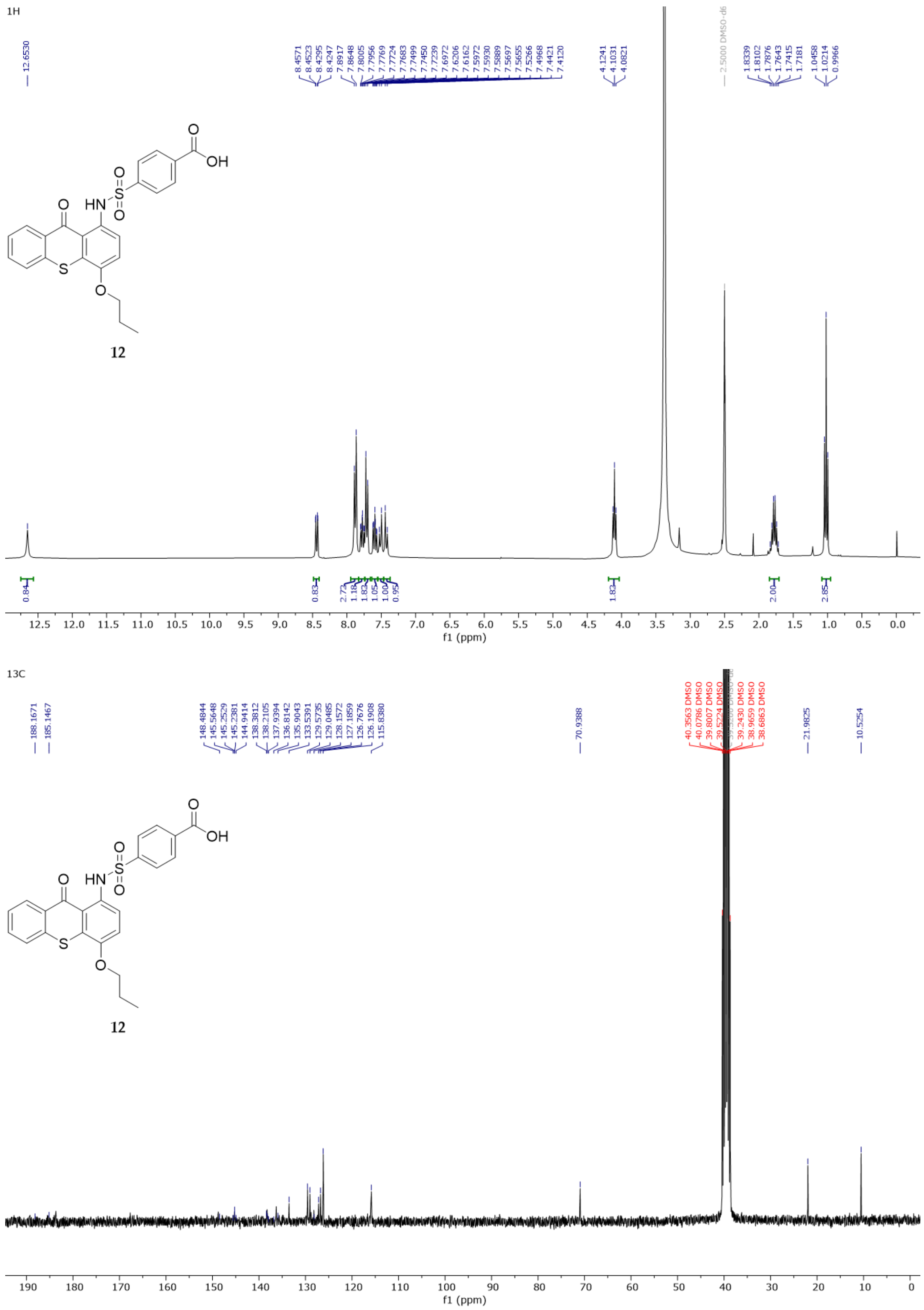

Figure S20. ^{1}H NMR (top, 300.13 MHz, DMSO) and ^{13}C NMR (bottom, 75.48 MHz, DMSO) for compound **12**.

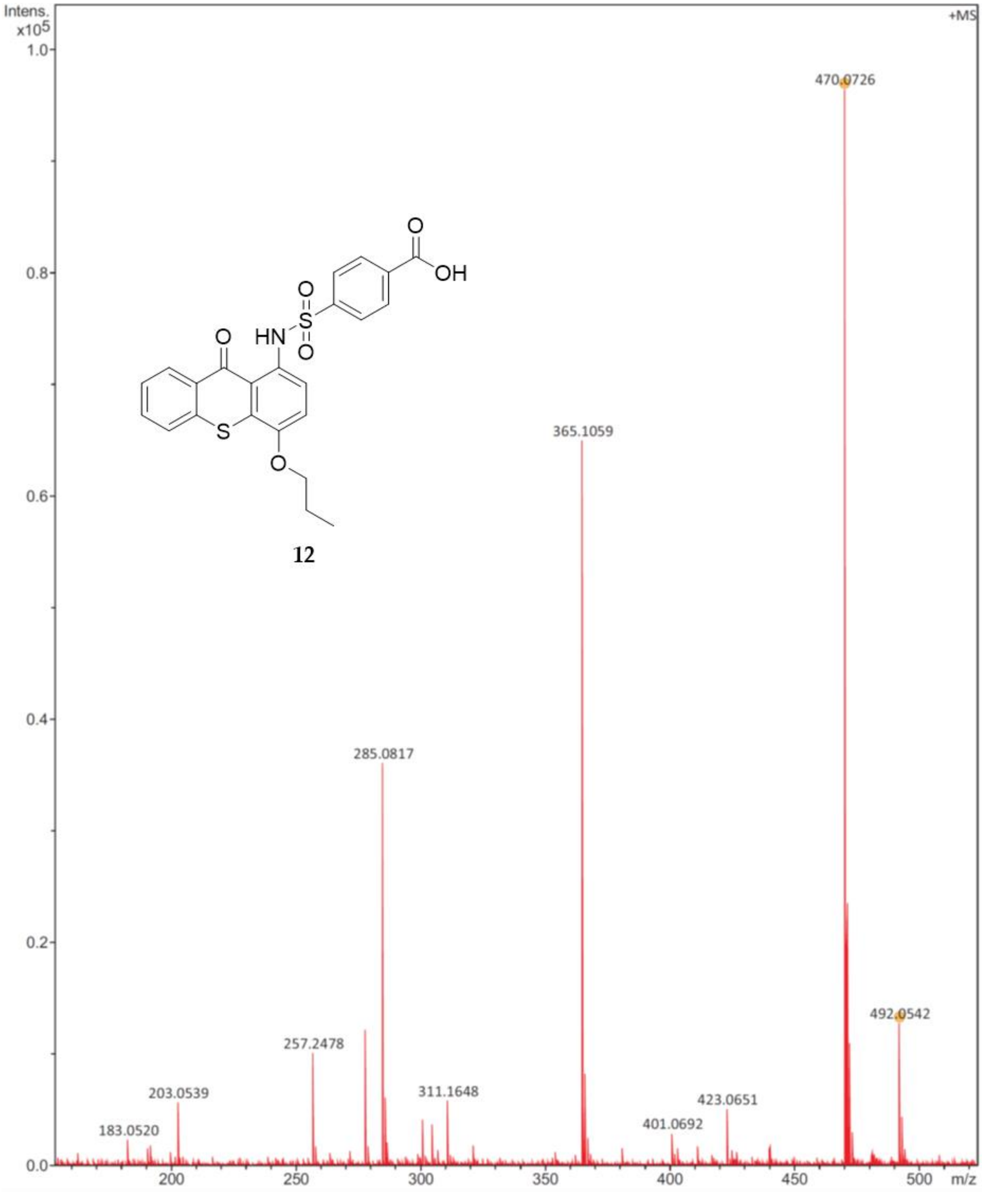

12

Meas. m/z	Formula	m/z	err [ppm]
470.0726	$C_{23}H_{20}NO_6S_2$	470.0732	-1.06

Figure S21. Electrospray ESI data for compound **12**.

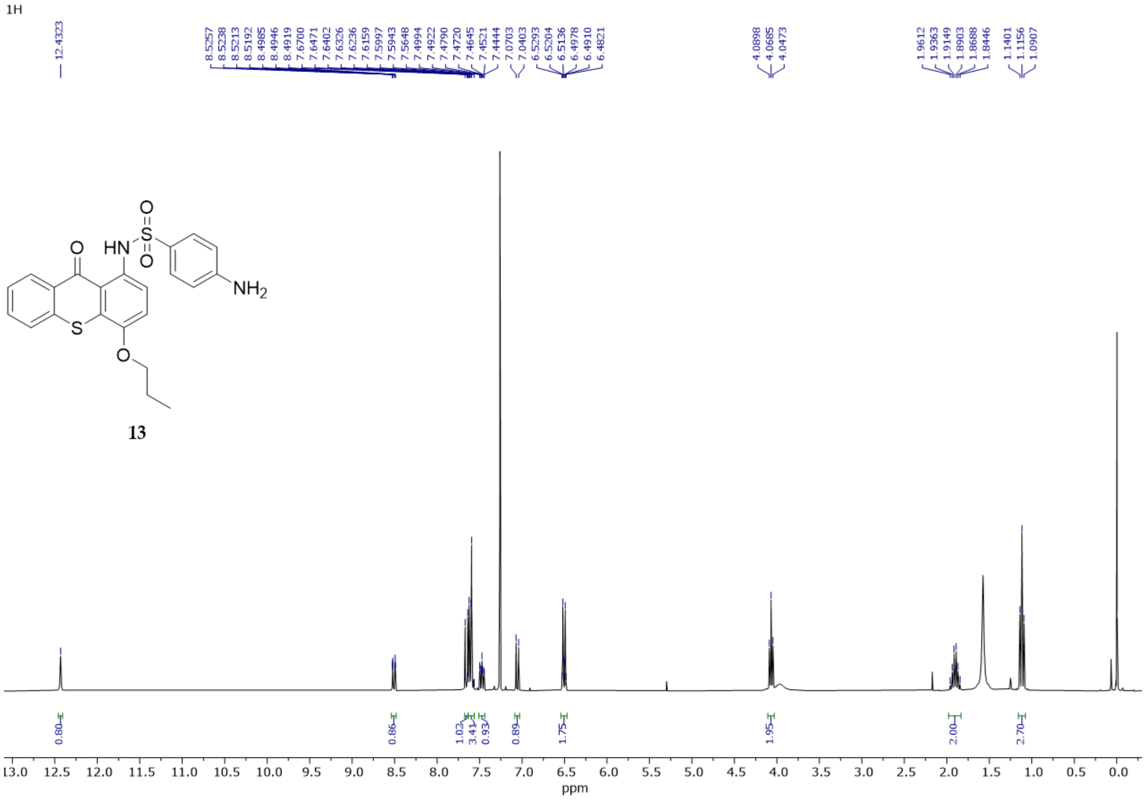

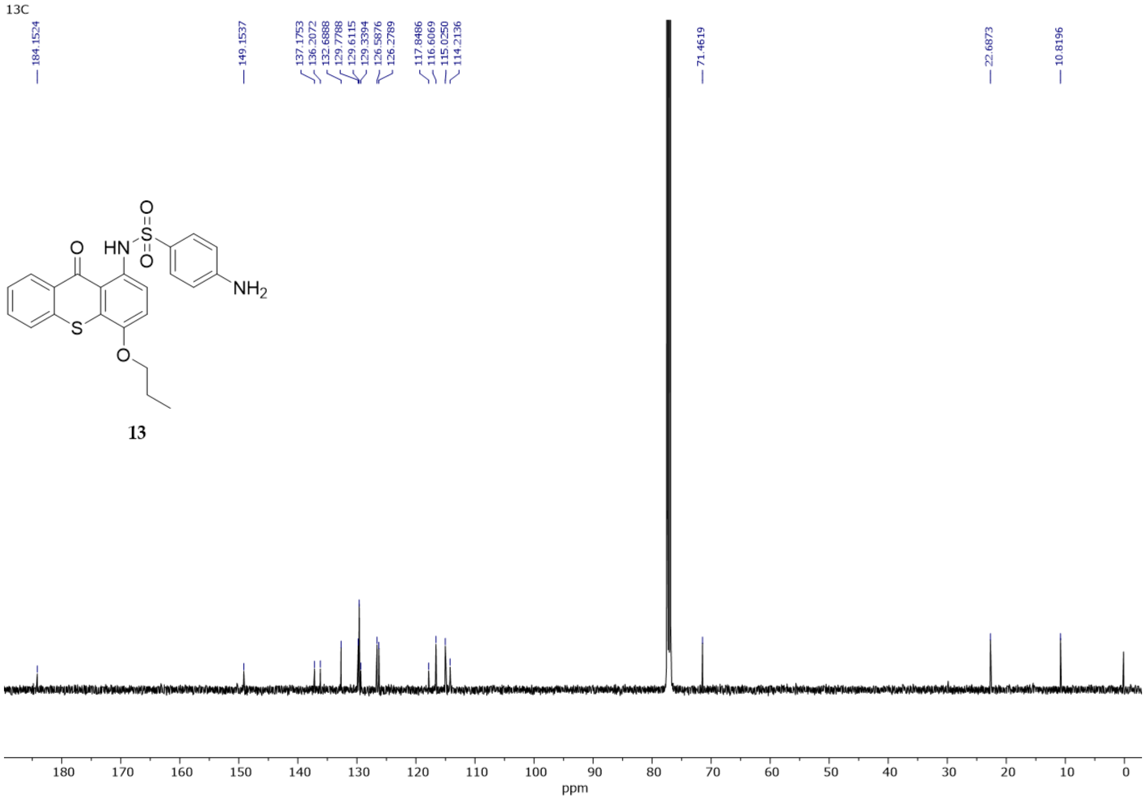

Figure S22. ^{1}H NMR (top, 300.13 MHz, $CDCl_3$) and ^{13}C NMR (bottom, 75.48 MHz, $CDCl_3$) for compound **13**.

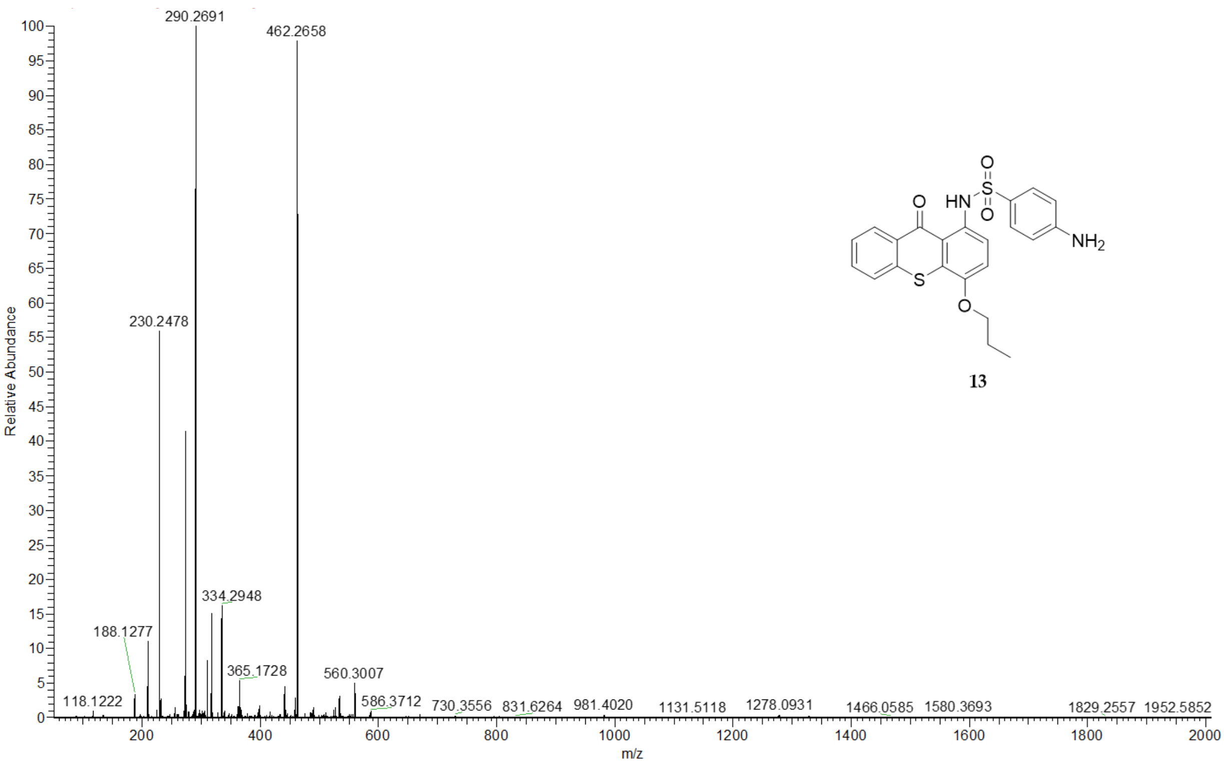

Meas. m/z	Formula	m/z	err [ppm]
462.2658	$C_{23}H_{19}NO_6S_2Na$	462.0683	427.43

Figure S23. Electrospray ESI data for compound **13**.

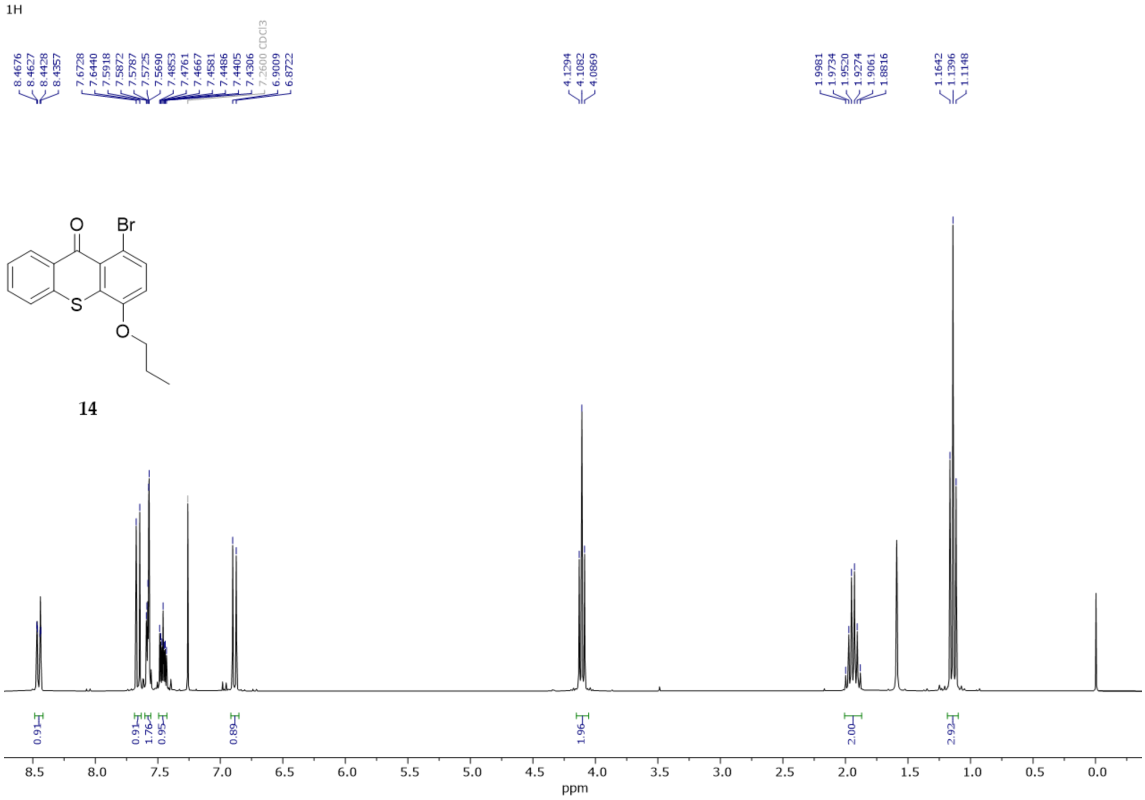

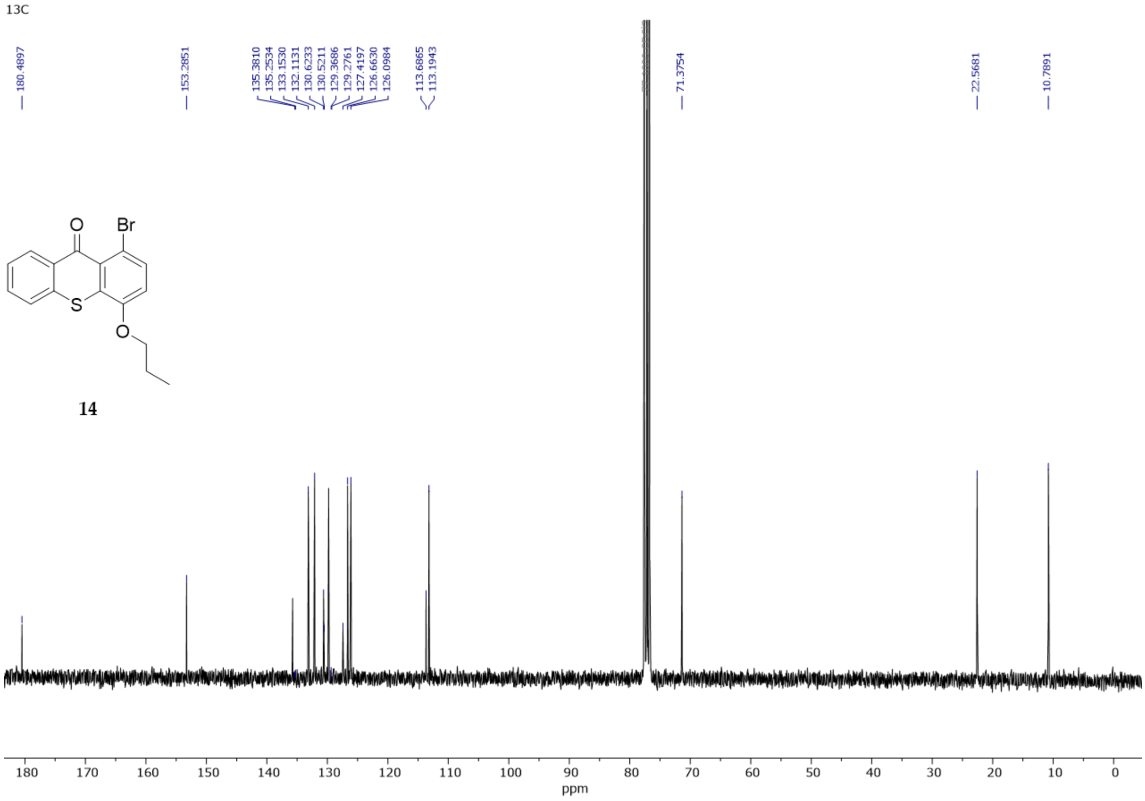

Figure S24. ^{1}H NMR (top, 300.13 MHz, $CDCl_3$) and ^{13}C NMR (bottom, 75.48 MHz, $CDCl_3$) for compound **14**.

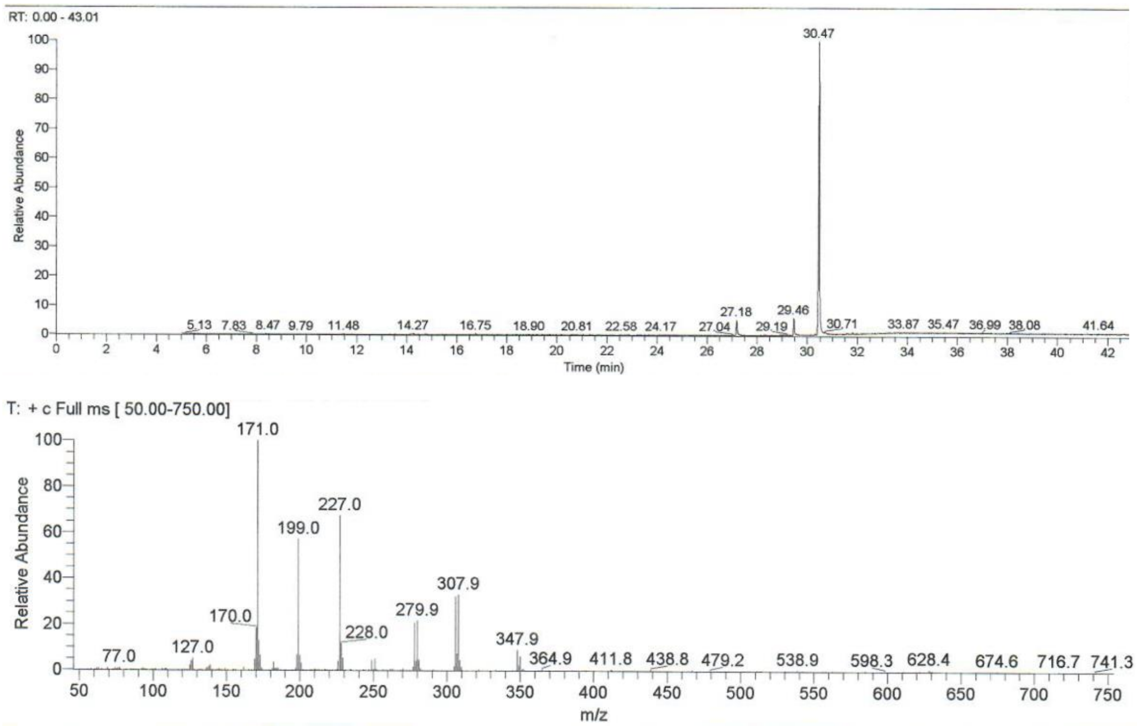

Figure S25. GC-MS spectra of compound **14**.

Table S1. Cytotoxicity of the selected compounds in NIH/3T3 cell line.

Compound	IC_{50} (μM) ± SD
**3**	>100
**8**	>100
**12**	64.47 ± 4.54
**13**	>100
**17**	>100
**Doxorubicin**	12.05 ± 0.81
